# Small-Molecule Ebselen Binds to YTHDF Proteins Interfering with the Recognition of *N*^6^-Methyladenosine-Modified RNAs

**DOI:** 10.1021/acsptsci.2c00008

**Published:** 2022-09-14

**Authors:** Mariachiara Micaelli, Andrea Dalle Vedove, Linda Cerofolini, Jacopo Vigna, Denise Sighel, Sara Zaccara, Isabelle Bonomo, Georgios Poulentzas, Emanuele Filiberto Rosatti, Giulia Cazzanelli, Laura Alunno, Romina Belli, Daniele Peroni, Erik Dassi, Shino Murakami, Samie R. Jaffrey, Marco Fragai, Ines Mancini, Graziano Lolli, Alessandro Quattrone, Alessandro Provenzani

**Affiliations:** † Department of Cellular, Computational and Integrative Biology, CIBIO, University of Trento, 38123Trento, Italy; ‡ Magnetic Resonance Center (CERM)—Department of Chemistry “Ugo Schiff”, University of Florence, 50019Florence, Italy; § Consorzio Interuniversitario Risonanze Magnetiche di Metalloproteine (CIRMMP), 50019Florence, Italy; ∥ Department of Physics, University of Trento, 38123Trento, Italy; ⊥ Department of Pharmacology, Weill Cornell Medicine, Cornell University, New York, New York10065, United States; # Department of Cellular, Computational and Integrative Biology, CIBIO, Mass Spectrometry Facility, University of Trento, 38123Trento, Italy

**Keywords:** YTHDF binders, *N*^6^-methyladenosine (m6A), ebselen, epitranscriptomic modulators, YTHDF structure, ebselen analogs

## Abstract

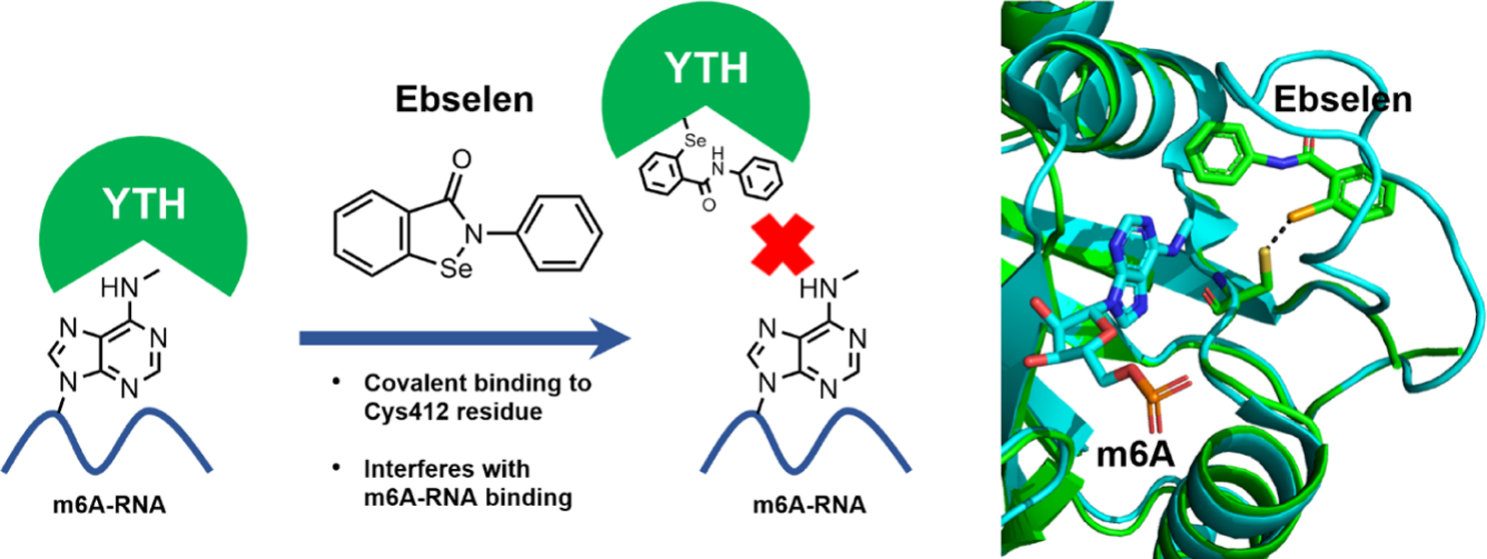

YTHDF proteins bind the *N*^6^-methyladenosine (m6A)-modified mRNAs, influencing their processing, stability, and translation. Therefore, the members of this protein family play crucial roles in gene regulation and several physiological and pathophysiological conditions. YTHDF proteins contain a hydrophobic pocket that accommodates the m6A embedded in the RRACH consensus sequence on mRNAs. We exploited the presence of this cage to set up an m6A-competitive assay and performed a high-throughput screen aimed at identifying ligands binding in the m6A pocket. We report the organoselenium compound ebselen as the first-in-class inhibitor of the YTHDF m6A-binding domain. Ebselen, whose interaction with YTHDF proteins was validated *via* orthogonal assays, cannot discriminate between the binding domains of the three YTHDF paralogs but can disrupt the interaction of the YTHDF m6A domain with the m6A-decorated mRNA targets. X-ray, mass spectrometry, and NMR studies indicate that in YTHDF1 ebselen binds close to the m6A cage, covalently to the Cys412 cysteine, or interacts reversibly depending on the reducing environment. We also showed that ebselen engages YTHDF proteins within cells, interfering with their mRNA binding. Finally, we produced a series of ebselen structural analogs that can interact with the YTHDF m6A domain, proving that ebselen expansion is amenable for developing new inhibitors. Our work demonstrates the feasibility of drugging the YTH domain in YTHDF proteins and opens new avenues for the development of disruptors of m6A recognition.

*N*^6^-Methyladenosine (m6A) is one of the most abundant and conserved RNA modifications. Collectively, these RNA modifications are called the epitranscriptome and are able to change the post-transcriptional fate of the target RNA.^1,2^ The discoveries of METTL3,^3^ which, assisted by a protein complex, is responsible for the synthesis of the majority of m6A modifications present on RNAs (writer), and of the demethylases FTO^4^ and ALKBH5 (erasers) lead to the understanding that this RNA modification is reversible and under the control of a regulated machinery. Genome-wide studies allowed the identification of the consensus m6A sequence RRACH (R = A or G, H = A, U, or C), located mainly near the stop codons or in the 3′ untranslated regions of mRNAs and in which the central adenosine is methylated.^5,6^ The RRACH motif is recognized by a specific class of proteins containing the YT521-B homology (YTH) domain and comprising in mammals three YTHDF proteins and two YTHDC proteins^7^ that differ in the homology in their YTH domain and their biological functions.^8^ YTH proteins are part of the epitranscriptome machinery and are devoted to recognize m6A RNAs and regulate the fate of target RNAs either in the nucleus or in the cytoplasm.^9−12^ The three YTHDF paralogs were initially ascribed with different biological functions, with YTHDF1 being a translation enhancer, YTHDF2 a facilitator of mRNA degradation, and YTHDF3 promoting either YTHDF1 or YTHDF2 function by direct interaction.^10,11,13^ Later on, the three paralogs have been proposed to have indistinguishable and redundant functions to control mRNA degradation by recruiting the mRNA deadenylation complex CCR4–NOT on m6A-containing mRNAs.^14−16^ Alteration of YTHDF protein expression levels, the hijacking of their function, or dysregulation of the m6A levels has been causatively correlated with the insurgence of some cancer types such as acute myeloid leukemia (AML), gastric carcinoma, hepatocellular carcinoma, and prostate cancer.^17−20^ In particular, high levels of YTHDF2 were observed in patient samples’ AML-derived leukemic stem cells (LSCs).^21^ YTHDF2 ablation increases the stability of the *TNFRSF*2 mRNA, which sensitizes LSCs to TNF-induced apoptosis, suggesting that inhibition of YTHDF2 could be beneficial for AML outcome. Indeed, the m6A epitranscriptome machinery recently emerged as a novel drug target, with initial effort focused on the FTO enzyme.^22^*In silico*-based screening allowed the identification of ligands of the METTL3-METTL14-WTAP complex that served as activators of the complex^23^ or inhibitors of METTL3.^24^ Notably, a nanomolar ligand of METTL3, STM2457, showed strong anticancer activity in cell lines and *in vivo* models of AML together with the ability to decrease m6A levels and modulate translation.^25^ No inhibitors of the YTHDF or YTHDC proteins have been still identified.

Here, we report the identification of a first-in-class inhibitor of the YTH domain of the YTHDF proteins. We selected this inhibitor, ebselen, through a small-molecule high-throughput screen (HTS). We characterized its binding mode to the YTH domain of the YTHDF proteins by X-ray and NMR structural studies and showed that ebselen is indeed able to bind to YTHDF proteins and interfere with the YTHDF–RNA interaction in cell lines. We also generated a series of ebselen-based analogs that interact with the YTH domain.

## Results

### Small-Molecule Screen Identifies the Organoselenium Compound Ebselen as a Binder of the YTH Domain

**Figure 1:**
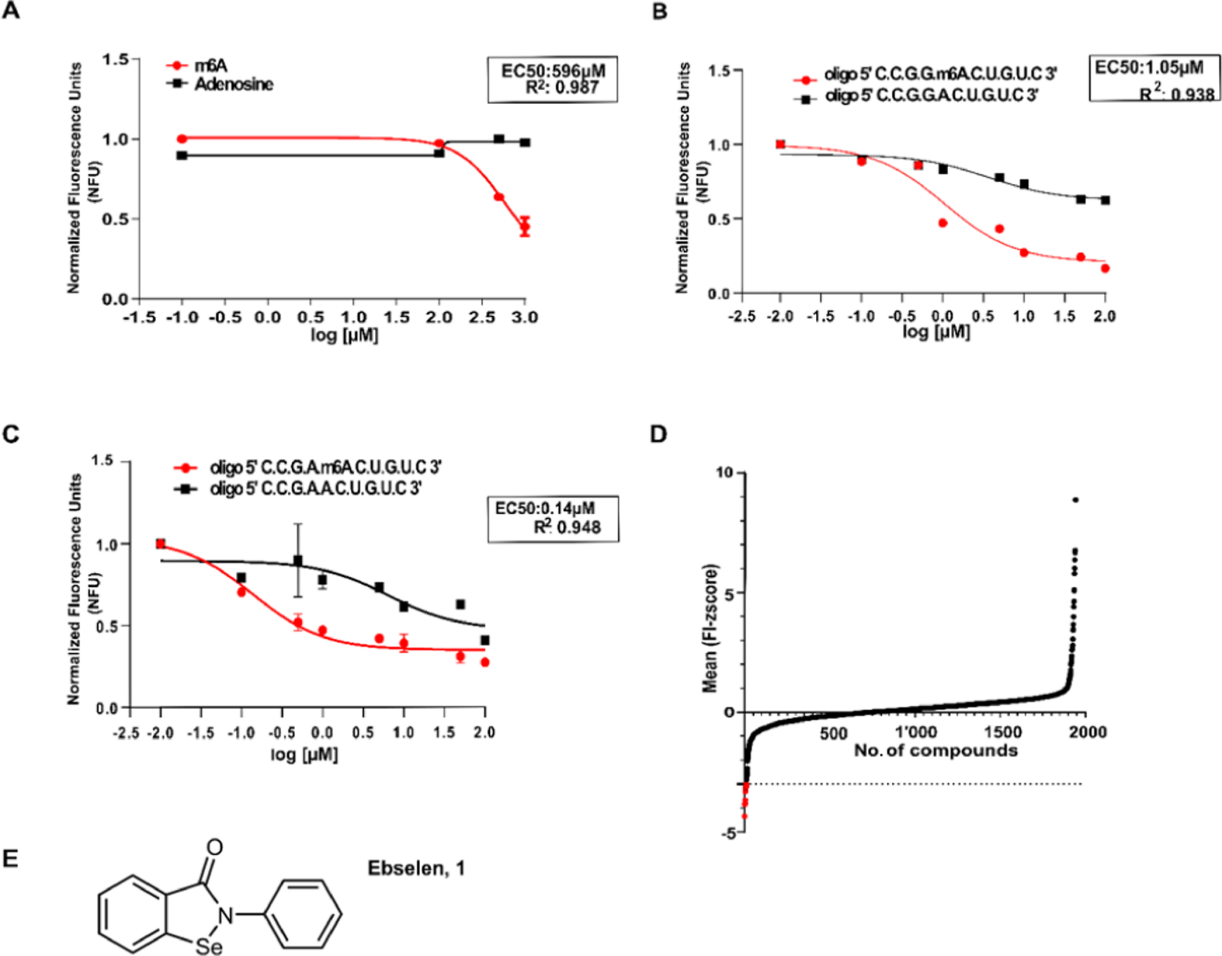
Organoselenium compound ebselen is an inhibitor of the YTH domain. (A) Binding specificity of the YTH domain toward m6A compared to adenosine. Data were fitted using a three-parameter nonlinear regression model *R*^2^ = 0.9870, EC_50_ = 596 μM. NFUs = normalized fluorescence units. (B) Fluorescence quenching by the ssRNA containing the GGm6ACU variant compared to the unmethylated one. Data were fitted using a three-parameter nonlinear regression model. *R*^2^ = 0.9386, EC_50_ = 1.05 μM. NFUs = normalized fluorescence units. (C) Fluorescence quenching by the ssRNA containing the GAm6ACU consensus variant compared to the unmethylated one. Data were fitted using a three-parameter nonlinear regression model. *R*^2^ = 0.9484, EC_50_ = 0.139 μM. NFUs = normalized fluorescence units. (D) Plot of progressive *Z*-score values of fluorescence intensity (FI) of 2560 compounds according to their quenching effect on the YTHDF1 domain. (E) Molecular structure of ebselen (**1**), the selected hit compound.

The YTH domains of YTHDF1, DF2, and DF3 share a common fold with a central β-sheet surrounded by five α-helices. Three conserved tryptophan residues largely define the hydrophobic pocket involved in m6A binding.^8,26^ Given the intrinsic fluorescence of the tryptophan residues present within the m6A-binding pocket, we investigated whether this feature could be used to set up an assay to screen for compounds able to interact with the YTH domain. We produced the recombinant YTHDF1 domain of the YTHDF1 protein spanning the amino acids 365–559, analyzed the purified fractions with Coomassie blue staining (Figure S1A), and evaluated whether an m6A molecule was able to quench the intrinsic tryptophan fluorescence. We observed a dose-dependent fluorescence quenching upon the addition of m6A (EC_50_ = 596 μM) but not upon adenosine addition, indicating that ligands able to bind to the pocket increase the hydrophobicity around the tryptophan sites, blocking tryptophan fluorescence.^27^ Similarly, fluorescence quenching was observed using two m6A ssRNA probes containing two variants of the consensus sequence [G(G/A)ACU]. We calculated an EC_50_ of 1.05 and 0.14 μM, respectively, for the two ssRNA probes, in line with literature data, with the GAm6ACU variant being, therefore, more potent.^8^ Notably, the two corresponding unmethylated ssRNAs could not quench tryptophan fluorescence, again indicating that fluorescence quenching is due to ligand binding (Figure 1A–C). The suitability of the assay for use in a small-molecule high-throughput screen (HTS) was evaluated by calculating the *Z*′ factor using the quenching effect of the m6A moiety. We obtained a value of 0.53, which indicated a robust assay suitable for HTS^28^ (Figure S1B). We performed a proof-of-principle screen, using a library of about 2560 small molecules, mainly composed of Food and Drug Administration (FDA)-approved drugs and biologically active compounds. We set up the screening by adding the protein (5 μM) in 384-well plates, together with the small molecules at a final concentration of 10 μM, using dimethyl sulfoxide (DMSO) as a negative control and the m6A as a positive control of quenching. Compounds were ordered according to the ability to modulate tryptophan quenching, expressed as *Z*-score (Figure 1D and Table S1). Hits were defined as the compounds able to induce a decrease of fluorescence higher than 3 standard deviations (SD) of the mean of the processed data (*p* = 0.0135, one-sided test).^29^ Four hits were identified. Hits were filtered *in silico*, to check for aggregation-prone molecules^30^ and to identify pan assay interference compounds (PAINS).^31^ Three out of four compounds were rejected by the *in silico* filtering for known aggregators or PAINS; ebselen was the only molecule that passed this quality check. Therefore, ebselen (2-phenyl-1,2-benzisoselenazol-3(2*H*)-one, **1**, Figure 1E), a small molecule in clinical trials for various diseases^32,33^ with anti-inflammatory, antioxidant, and cytoprotective activities, was chosen for further characterization.^34,35^

### Ebselen Binds to the YTHDF YTH Domain and Disrupts the YTH/RNA Interaction

**Figure 2:**
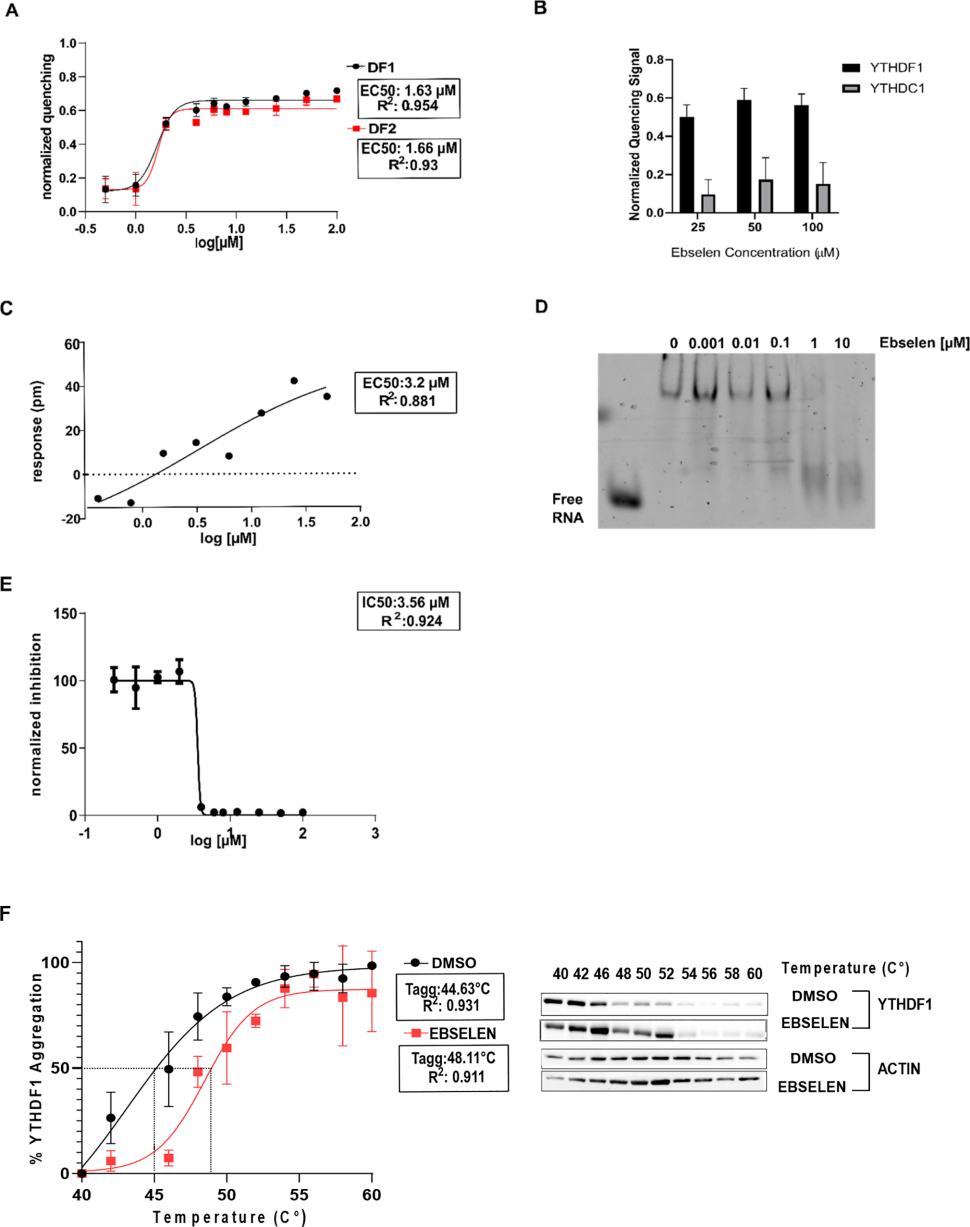
Organoselenium compound ebselen is an inhibitor of the YTH domain and can bind YTHDF1 in cells. (A) Dose–response curves of increasing amounts of ebselen added to the YTHDF1 and YTHDF2 protein domains were obtained with the tryptophan quenching assay. Data were fitted with a four-parameter nonlinear regression model, *R*^2^ = 0.954 and 0.93, EC_50_ of 1.63 and 1.66 μM for the YTHDF1 and YTHDF2 YTH domains, respectively. (B) Ebselen cannot reduce tryptophan fluorescence of the YTH domain of the YTHDC1 protein. (C) Dynamic mass redistribution (DMR) assay to evaluate ebselen binding at equilibrium. Measurements were performed before (baseline) and after (final) compound addition. The response (in picometers (pm)) was measured by subtracting the baseline output from the final output signals. The output signal for each well was obtained by subtracting the signal of the protein-coated reference area from the signal of the uncoated area. The data were fitted to a sigmoidal function using a four-parameter logistic (4PL) nonlinear regression model: *R*^2^ = 0.8147, EC_50_ = 3.22 μM. (D) REMSA shows the inhibitory effect of the candidate drug ebselen on the RNA-binding activity of the YTH domain of YTHDF1, starting from 1 μM. (E) Determination of the IC_50_ value of the ebselen molecule with the AlphaScreen assay, using nonlinear regression fits of the data according to a four-parameter nonlinear regression model: *R*^2^ = 0.9844, IC_50_ = 3.565 ± 0.009 μM. (F) *T*_agg_ curves and cellular thermal shift assay (CETSA) Western blots for YTHDF1 in HEK293T cells in the presence of DMSO and 50 μM ebselen. Ebselen causes a shift of 4 °C in the *T*_agg_ of YTHDF1 in HEK293T cells. The CETSA data are expressed as mean ± SD (*n* = 3 independent assays); relative band intensities were fitted using a sigmoidal (variable slope) curve fit. *T*_agg_ values are determined where there is 50% of YTHDF1 aggregation. DMSO: *R*^2^ = 0.9416, *T*_agg_ = 44.63 °C ± 2. Ebselen: *R*^2^ = 0.9113, *T*_agg_ = 48.11 °C ± 1.36 (Δ*T*_agg_ = 3.48 °C, **p = 0.0044, two-way analysis of variance (ANOVA)).

To understand whether ebselen can discriminate among the YTH domains, we performed dose–response experiments adding increasing amounts of ebselen to the YTH domains of the YTHDF1 and YTHDF2 proteins using the tryptophan quenching assay. For this reason, we also produced the recombinant YTHDF2 domain comprising the amino acids 380–579 (Figure S1C). The EC_50_ of 1.63 and 1.66 μM, respectively, for the YTHDF1 and YTHDF2 domains, suggested that ebselen cannot discriminate between the two proteins (Figure 2A). Notably, ebselen did not interact with the YTH domain of the YTHDC1 protein (Figures 2B and S2). Therefore, we focused on YTHDF1 for the subsequent experiments. We first measured the EC_50_ of interaction between the protein and the recognized ssRNA by label-free dynamic mass redistribution (DMR), obtaining a value of 63.76 nM (Figure S3), in line with other reports (56 nM^36^). The DMR analysis also revealed a direct interaction between ebselen and the YTHDF1 domain (Figure 2C). We derived a dose–response curve and calculated an EC_50_ value of 3.22 μM, in agreement with the data obtained by the tryptophan quenching assay. Ebselen interfered with the RNA-binding activity of the YTHDF1 YTH domain starting from 0.1 μM, as evaluated by an RNA electrophoretic mobility shift assay (REMSA; Figure 2D). We also investigated the ability of ebselen to inhibit the formation of the protein–RNA complex in saturation-binding conditions through the AlphaScreen technology. We calculated the IC_50_ values on fitted AlphaScreen saturation curves in the presence of ebselen at different concentrations in the μM range (0–100 μM). We obtained an IC_50_ of 3.565 ± 0.009 μM (Figure 2E). Taken together, these data suggest that ebselen interacts with the YTH domain of the YTHDF proteins and disrupts the binding with methylated consensus ssRNA probes, likely modifying the conformation of the protein according to the tryptophan quenching data.

To investigate *in cellulo* the inhibitory activity of ebselen toward the RNA–protein complex of the YTH domain, we checked ebselen binding to YTHDF1 in cell culture by the cellular thermal shift assay (CETSA).^37^ This assay assumes that the temperature of the unfolding of a specific protein, the so-called aggregation temperature (*T*_agg_), can be modified by binding a small molecule, causing a thermal shift. We treated HEK293 cells with 50 μM ebselen for 3 h, collected the cells, divided the pellets, and heated them at different temperatures. Aggregated materials were eliminated by centrifugation, and the soluble YTHDF1 was measured by Western blot. Indeed, ebselen significantly increased the YTHDF1 *T*_agg_ to 3.48 °C, indicating that it binds to the protein inside cells (Figure 2F).

### Ebselen Inhibits the Interaction of the YTHDF2 YTH Domain with Bound mRNAs in Cells

**Figure 3:**
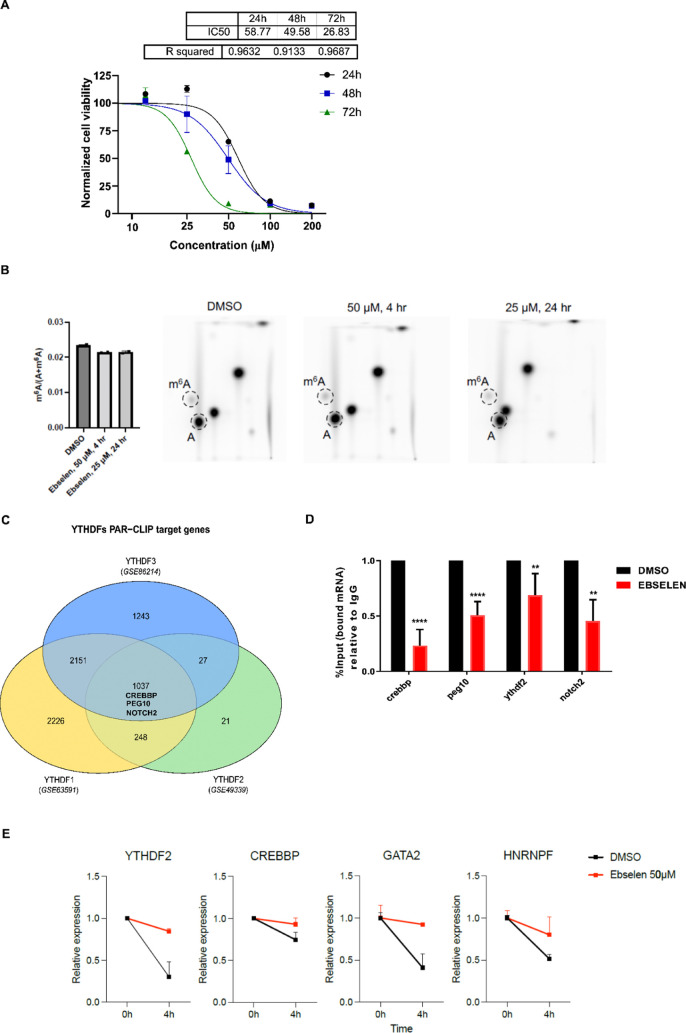
Ebselen affects the viability of prostate cancer cells and interferes with the RNA-binding ability of YTHDF2. (A) Cell viability was determined with an OZblue kit after 24, 48, and 72 h of treatment with different ebselen concentrations (1, 5, 10, 25, 50, 100, and 200 μM). Data were normalized and fitted with a four-parameter nonlinear model (24 h: *R*^2^ = 0.9632, IC_50_ = 58.77 μM; 48 h: *R*^2^ = 0.9133, IC_50_ = 49.58 μM**;** and 72 h**:***R*^2^ = 0.9687, IC_50_ = 26.83 μM). (B) m6A levels in poly(A) purified mRNA were quantified by two-dimensional (2D) thin-layer chromatography (TLC, see the Materials and Methods section). The quantification is shown. *n* = 2 independent experiments; error bars, s.e. (C) Diagram showing the selected targets derived from the intersection of three different PAR-CLIP datasets on GEO (GSE63591 for YTHDF1, GSE49339 for YTHDF2, and GSE86214 for YTHDF3). *PEG10*, *NOTCH2*, and *CREBBP* were selected. (D) Ribonucleoprotein immunoprecipitation (RIP) assay followed by quantitative real-time quantitative polymerase chain reaction (qRT-PCR). PC-3 cells were treated for 24 h with DMSO (control) and 25 μM of ebselen. Subsequently, cells were lysed, RNA precipitated with a YTHDF2 antibody, and the corresponding IgG isotype as a negative control. Changes in the bound mRNA were quantified through qRT-PCR, normalizing the values to the 18S housekeeping RNA and dividing the IgG and the YTHDF2 fraction values by the values obtained from the input, corresponding to the 1% of RNA used in the RIP. Data are plotted relative to the DMSO sample, the control. Data are presented as means ± SD of a biological triplicate (**p = 0.0021, ****p < 0.0001, *versus* control). (E) Stability of m6A-mRNAs was determined by quantifying mRNA levels before and after 4 h of actinomycin D treatment. Shown is the change in mRNA levels compared to the condition of no actinomycin D treatment. The increase in mRNA stability is present upon ebselen treatment compared to the DMSO condition. *n* = 2 replicates; error bars, s.e.

To evaluate whether part of the ebselen biological activity is associated with the modulation of the YTH domain of YTHDF proteins, we decided to focus on the prostate cancer cell line PC-3. The PC-3 cell line was chosen because YTHDF2 has shown to be involved in cancer development and its expression level is correlated with the poor prognosis of prostate cancer patients.^20^ To assess which concentration could be feasible to perform further experiments, we evaluated the molecule’s toxicity by treating cells with different concentrations for 24, 48, and 72 h. IC_50_ values were calculated for each time point (Figure 3A), and the compound’s toxicity increased with increasing hours of treatment. No changes in the expression level of the most relevant proteins of the epitranscriptome apparatus were observed (Figure S4A–C). We then evaluated the level of overall m6A methylation in the exome of PC-3 cells and observed no significant changes in the 24 h time frame (Figure 3B).

We then performed a ribonucleoprotein immunoprecipitation (RIP) assay in PC-3 cells, checking for the mRNA binding capacity of YTHDF2. We treated cells with a subtoxic dose of ebselen (25 μM) for 24 h and with DMSO as the control. After cell lysis and coprecipitation of RNA with both the YTHDF2 antibody and the relative IgG isotype as a control, we performed real-time quantitative PCR (qRT-PCR) to quantify the expression level of target mRNAs in each sample and to evaluate their depletion after treatment. To decide which target mRNAs to focus on, we ranked the enriched target mRNAs that emerged from three YTHDF PAR-CLIP datasets in GEO (GSE63591 for YTHDF1, GSE49339 for YTHDF2, and GSE86214 for YTHDF3)^11,38,39^ and identified the mRNAs present in all three datasets (Figure 3C and Table S2). *PEG10* and *NOTCH2* were in the top-ranked 100 mRNAs of the YTHDF1 dataset, present in the YTHDF2 dataset, and in the top 10 of the YTHDF3 dataset and thus were chosen as target mRNAs. In addition, we also selected *CREBBP*, which is considered a *bona fide* YTHDF2 target gene, at least in HeLa cells.^10^ All of the genes were significantly enriched in the YTHDF2-bound mRNA fractions. Ebselen treatment resulted in interference with YTHDF2 and its targets, as suggested by the decrease of the mRNA levels compared to control samples (Figure 3D). The stabilities of *CREBBP* and additional known methylated genes in prostate cancer cell lines^40^ (*YTHDF2*, *GATA2*, and *HNRNPF*) were assessed after 4 h of co-treatment of ebselen and actinomycin D. In these conditions, ebselen treatment increased the stability of methylated genes (Figure 3E).

These data indicate that ebselen can bind to YTHDF2 in cell culture conditions, interferes with its ability to bind at least some of its target mRNAs, and partially promotes their stability

### Ebselen Binds Covalently or Interacts Reversibly with the YTH Domain of the YTHDF1 Protein Depending on the Reducing Environment

**Figure 4:**
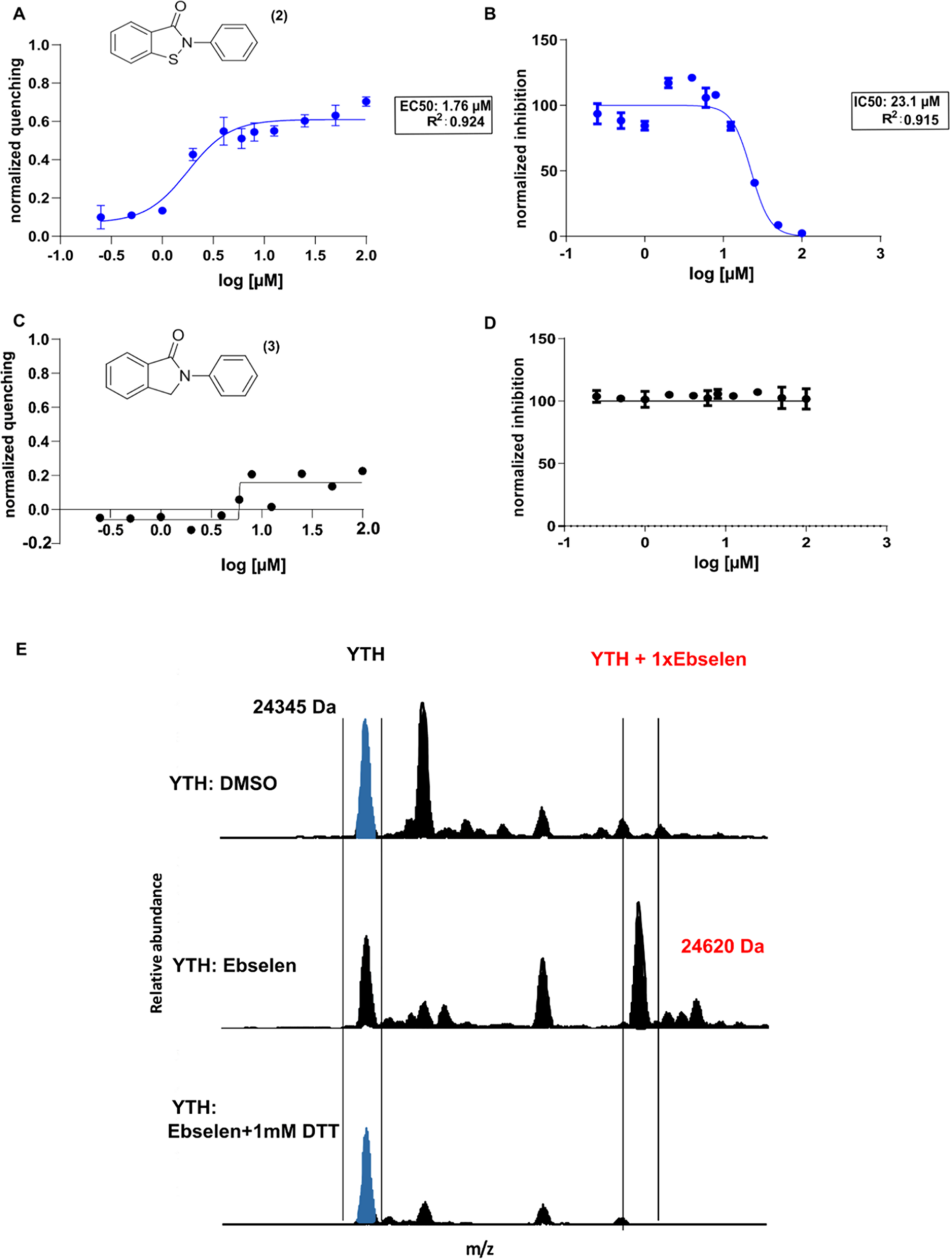
Ebselen covalently binds to the YTH domain of the YTHDF1 protein, and the substitution of the Se atom with S maintains its ability to interact with the domain and disrupt its RNA-binding ability. (A) Dose–response curve obtained with increasing concentrations of ebsulfur in the tryptophan quenching assay. Data were fitted with a four-parameter nonlinear regression model, *R*^2^ = 0.924 and EC_50_ = 1.76 ± 0.02 μM. (B) Determination of the IC_50_ value of the ebsulfur molecule with the AlphaScreen assay, using nonlinear regression fits of the data according to a four-parameter nonlinear regression model: *R*^2^ = 0.915, IC_50_ = 23.14 ± 0.08 μM. (C, D) Compound **3** failed to generate a dose–response curve in the tryptophan quenching assay and to disrupt the RNA-binding ability of the YTH domain in the AlphaScreen assay. (E) Mass spectra of the YTH domain of YTHDF1 (4RCJ structure in PDB), alone or in the presence of ebselen (50 μM) with or without 1 mM DTT.

Ebselen interacts with thiols and forms selenium sulfide bonds^41^ with many cysteine-rich proteins, including SOD1,^42^ the hepatitis C virus nonstructural protein 3 helicase NS3,^43^ and the SARS-CoV-2 virus proteases.^44,45^ It has been suggested that ebselen is a cysteine modifying agent that behaves as a reversible or an irreversible binder depending on concentration, reducing conditions of the reaction environment, and incubation time.^41^ Therefore, we investigated the relevance of the selenium atom and whether the interaction with the YTH domain is reversible or due to covalent binding. The substitution of the selenium atom with a sulfur atom (ebsulfur, compound **2**) maintained both capacities (Figure 4A,B). In contrast, the substitution with a CH_2_ unit (2-phenylisoindolin-1-one, compound **3**) completely abolished the compound’s ability to bind the YTH domain and disrupt its interaction with its target RNA (Figure 4C,D). We incubated 5 μM of the recombinant YTH domain of the YTHDF1 protein with 50 μM of ebselen for 60 min and removed the unbound compound through a 7 kDa molecular weight cutoff (MWCO) filter. The samples were then subjected to electrospray ionization mass spectrometry (ESI-MS) analysis using direct infusion. The YTH domain displayed a prominent peak with a mass of 24 345 Da, whereas treatment with ebselen induced a mass shift of 274.96 Da, consistent with mass increases of one bound ebselen molecule (Figure 4E). The recombinant YTH domain was then incubated with ebselen in the presence of 1 mM dithiothreitol (DTT), which reduces sulfur–selenium bonds, and analyzed by MS. In this reducing condition, the mass matched that of the unmodified YTH domain of YTHDF1, confirming that the addition of DTT reduced the selenylsulfide bond between ebselen and its target (Figure 4E). These data show that ebselen binds covalently to the YTH domain but can dissociate depending on the environment-reducing state.

### Ebselen Binds Covalently to Cys412 and Nearby the m6A-Binding Pocket of the YTHDF1 Protein

**Figure 5:**
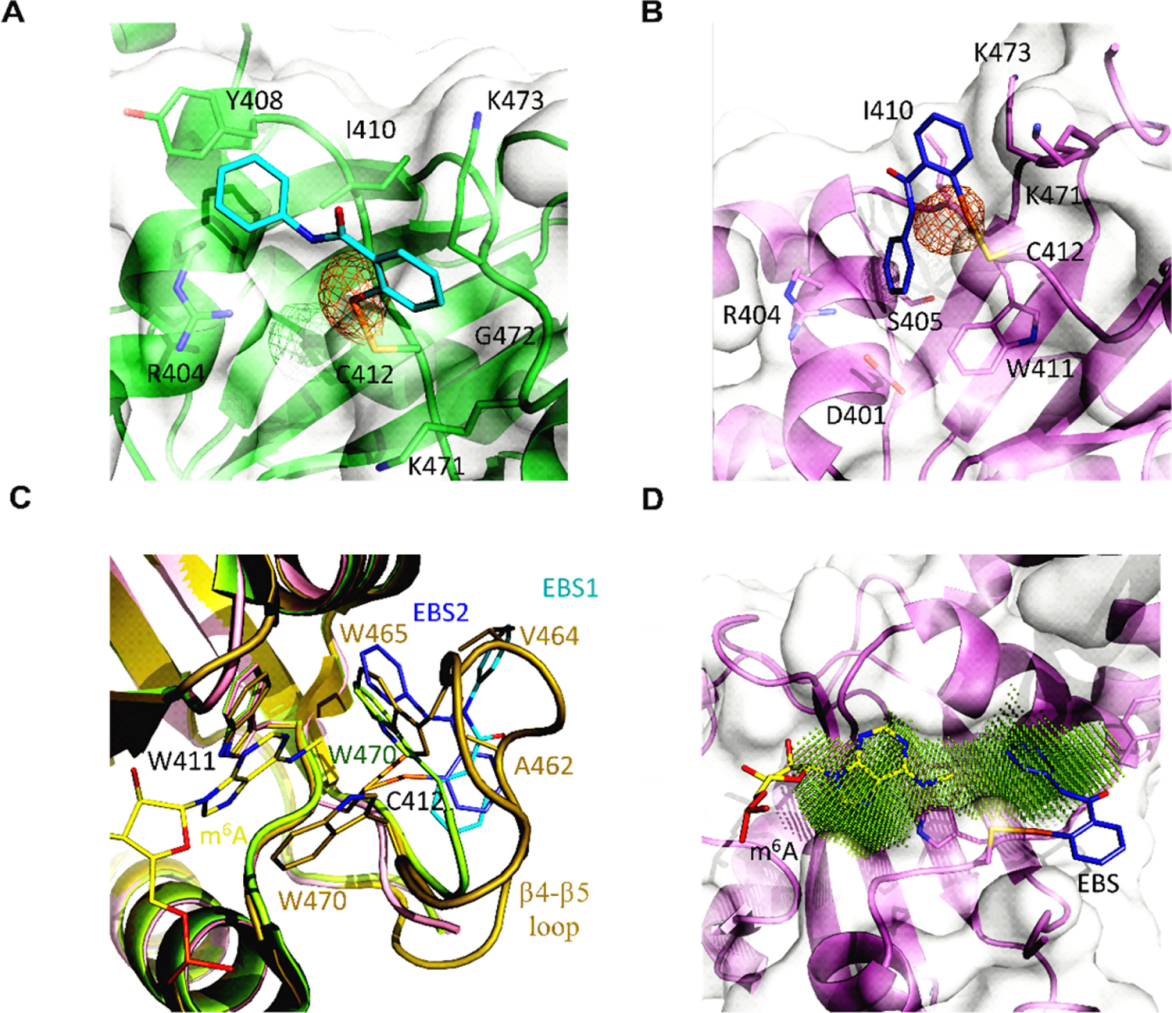
Ebselen interferes with the correct organization of the m6A-binding pocket. (A, B) Ebselen binds the YTHDF1 YTH domain adopting different poses; the protein matrix is shown in green with ebselen in cyan for pose 1, while for binding mode 2, the YTH domain is in violet with the ebselen molecule in slate. The selenium anomalous map is contoured at 3.5σ and shown in orange. (C) In the holo 4RCJ structure (dark yellow), the β4−β5 loop organizes its structure on the bound m6A (yellow); in the apo 4RCI structure (lime), the loop is disordered, but Trp470 inserts into the binding pocket; and ebselen (color code: cyan for pose 1, slate for pose 2) binding is incompatible with the m6A-binding-competent conformation. (D) Due to Trp465 or Trp470 displacement, ebselen enlarges the m6A pocket; the druggable pocket has been identified with DoGSiteScorer.^46^

To further study the ebselen binding and mode, the YTH domain of the YTHDF1 protein was co-crystallized with ebselen, and X-ray diffraction data were collected at the Se absorption edge to locate the Se atom unambiguously. The Se atom appeared to be involved in a covalent bond with Cys412 (Figures 5A,B and S5 and Table S3). The ebselen molecule sits on a very shallow groove with one side resting on the locally hydrophobic protein matrix, while the other face remains solvent-exposed. However, in the two protein chains in the asymmetric unit, ebselen preferentially adopts two slightly different poses. In the first case (Figure 5A), the selenobenzene moiety almost inserts between the aliphatic segments of the Lys471 and Lys473 side chains while also contacting the intervening Gly472; the *N*-phenyl amide group stacks to Tyr408 concurrently interacting with the Arg404 and Ile410 side chains. In the other pose (Figure 5B), while the selenobenzene group maintains similar interactions with a minor ring rotation (32°), the *N*-phenylformamide moiety inserts more deeply into the groove in van der Waals contact to Asp401, Arg404, Ser405, Ile410, and Trp411.

Electron density is missing for a relevant part of the extended β4−β5 loop (residues 460–469), which is then flexible and disordered. This is consistent with the deposited apo structure (PDB code 4RCI). In contrast, in the holo structure (PDB code 4RCJ) complex with a short m6A-containing oligoribonucleotide, the loop folds on top of the ligand and constitutes part of the binding interface (Figure 5C). The binding of m6A then induces a conformational change with Trp465 becoming part of the Trp cage (together with Trp411 and Trp470), trapping the ligand in a deep pocket located close to Cys412, but on the opposite side with respect to the ebselen binding groove.

Although binding at a site different from the m6A pocket, the ebselen molecule impedes the conformational rearrangement of the β4−β5 loop into the m6A-binding-competent conformation. The selenobenzene ring in both ebselen poses clashes with YTHDF1 residues Ser461–Ala462 in the m6A-bound structure, while the *N*-phenylformamide group either collides with Trp465 or Val464 (Figure 5C). Notably, in the apo 4RCI structure, Trp470 shifts from its m6A-binding position and substitutes Trp465, possibly outlining the m6A-binding site that fully reorganizes upon substrate binding; this conformation is also incompatible with ebselen binding.

Finally, ebselen displacement of both Trp465 and Trp470 generates a unique pocket extending to the m6A-binding region that can be exploited by ebselen derivatives with improved potency (Figure 5D).

### Ebselen Interacts Noncovalently with the m6A-Binding Pocket in Reducing Conditions

To further investigate the ligand–protein interaction in reversible conditions, the resonances of the YTHDF1 YTH domain were assigned. The protein sample was titrated with ebselen, in the presence of β-mercaptoethanol (10 mM) as a reducing agent, to prevent the covalent bond formation. The titration was monitored by NMR to map at atomic resolution the ligand-binding site on the protein surface. Increasing amounts of the ligand (12.5, 25, 50, 100, and 200 μM) were added to the protein solution during the NMR titration, and 2D ^1^H–^15^N heteronuclear single quantum coherence (HSQC) spectra were acquired.

**Figure 6:**
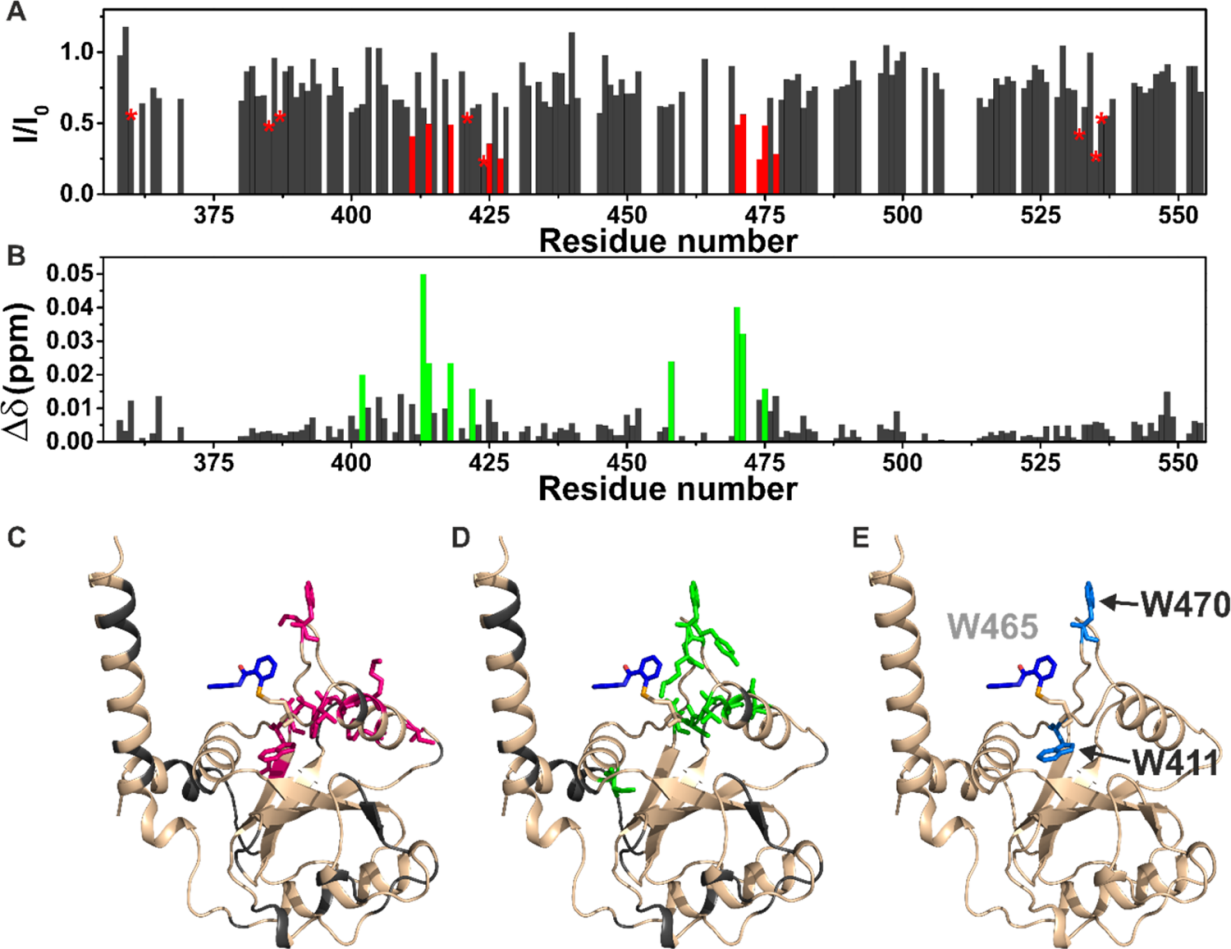
Ebselen specifically interacts with the hydrophobic pocket of the YTH domain. (A) Plot of the decreases in signal intensity of the YTHDF1 YTH domain (100 μM) in the presence of the ligand ebselen (50 μM); the residues exhibiting the most significant decreases are highlighted in red. The stars indicate residues with a significant decrease in signal intensity but overlapping in the NMR spectra. (B) Plot of the chemical shift perturbations (CSPs) of the YTHDF1 YTH domain (100 μM) in the presence of the ligand ebselen (50 μM), evaluated according to the formula ; the residues exhibiting the largest CSP are highlighted in green. (C) Cartoon representation of the YTHDF1 YTH domain in complex with ebselen displayed as blue sticks (PDB code: 7PCU) highlighting in magenta the residues exhibiting the most significant decreases in signal intensity; in gray, the unassigned residues. (D) Cartoon representation of the YTHDF1 YTH domain in complex with ebselen displayed as blue sticks (PDB code: 7PCU) highlighting in green the residues exhibiting the largest CSP; in gray, the unassigned residues. (E) Cartoon representation of the YTHDF1 YTH domain in complex with ebselen displayed as blue sticks (PDB code: 7PCU) with the three tryptophan of the binding pocket highlighted in blue on the protein in complex with an m6A consensus ssRNA, displayed as yellow sticks. The tryptophan residue W465 was not assigned in the NMR spectra as well as in a region with low electron density in the X-ray structure.

In the presence of substoichiometric concentrations of the ligand, a decrease in signal intensity was observed for some protein residues (W411, T414, N418, K419, F425, C427, G459, W470, F474, D475, Q477, F536, and A537, Figure 6A,C) as expected for a ligand with an affinity constant in the low micromolar range, which is in an intermediate exchange regime on the NMR time scale. Few of these residues and some of their neighboring amino acids (I402, S413, T414, N418, D422, Y458, W470, K471, and D475, Figure 6B,D), however, also experienced a tiny chemical shift perturbation (CSP). Interestingly, among the residues experiencing the most significant decreases in signal intensity, we found two (W411 and W470) out of three of the tryptophan residues present inside the binding pocket of the domain (Figure 6E). Unfortunately, the assignment of the third tryptophan residue (W465) is missing. Therefore, no information can be retrieved on this residue. To investigate how ebselen interferes with the binding to an m6A RNA substrate, a protein sample was titrated with increasing amounts of the RNA consensus oligoribonucleotide. In the presence of substoichiometric concentrations of the m6A–RNA (40 μM), a decrease in signal intensity was observed for some protein residues (H365, D400, D401, I402, S409, I410, W411, S413, T414, G417, N418, S439, M452, D457, G459, W470, F474, Q477, V484, and R506) (Figure S6A–C). In the presence of stoichiometric amounts of the m6A–RNA substrate, new peaks corresponding to the protein in complex with the RNA were visible in the spectrum. The protein–RNA interaction was strong and in the slow exchange regime on the NMR time scale. Collectively, the NMR data prove that the residues experiencing effects in the presence of ebselen are located in the pocket responsible for the binding to the methylated RNA (Figure 6), indicating that the interaction with this ligand occurs specifically in this hydrophobic pocket. Interestingly, this interaction occurs with micromolar affinity regardless of the selenium sulfide bonds. These findings also agree with the fluorescence data and explain the quenching of intrinsic protein fluorescence observed after interaction with the ebselen ligand. Therefore, ebselen interacts with the m6A-binding pocket either in reducing or oxidizing conditions, but the presence of the selenium atom appears to be necessary to drive the binding.

### Synthesis of First-In-Class, Ebselen-like YTH Domain Ligands

**Figure 7:**
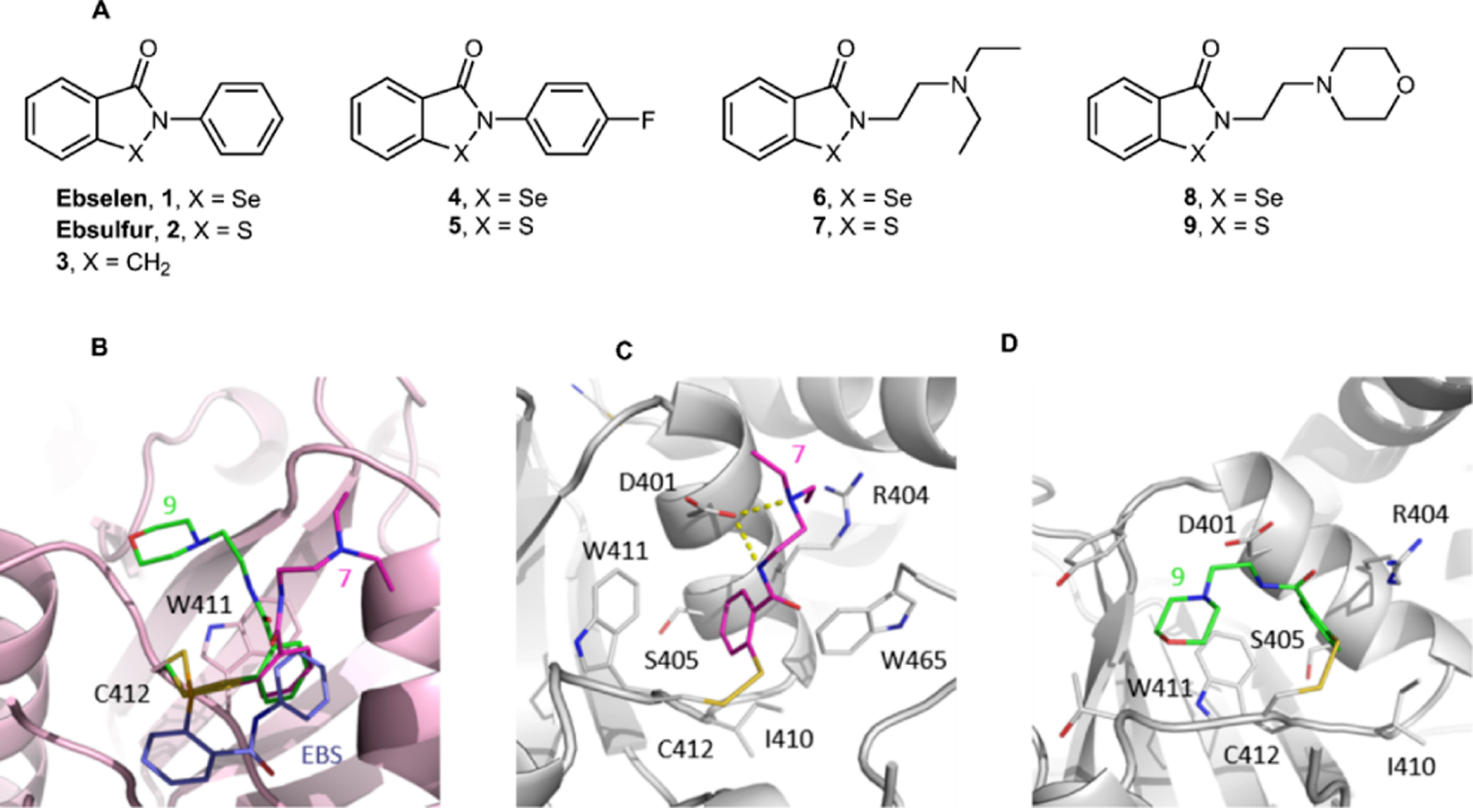
Molecular structure of new synthetic ebselen and ebsulfur analogues and co-crystal structures of compounds **7** and **9** with the YTHDF1 YTH domain. (A) Molecular structures of ebselen and ebsulfur analogues. (B) Compound **7** (magenta) and **9** (green) interact with the YTH domain through a disulfide bond with Cys412 but are oppositely directed with respect to ebselen (violet). (C, D) Detailed interaction of compound **7** and **9** with the YTH protein matrix.

Ebselen was shown to establish a covalent interaction with the YTH domain forming a reversible selenium sulfide bond with a Cys residue in the hydrophobic pocket. We produced a series of ebselen analogues to enhance the interaction of the ligand with the YTHDF YTH domain. Based on the well-known similarity of selenium and sulfur in their physicochemical properties such as ion radii, redox potentials, and electronegativity, we designed and synthesized ebsulfur (**2**) and compounds **4**–**9** (Figure 7A).^47^

**Scheme 1:**
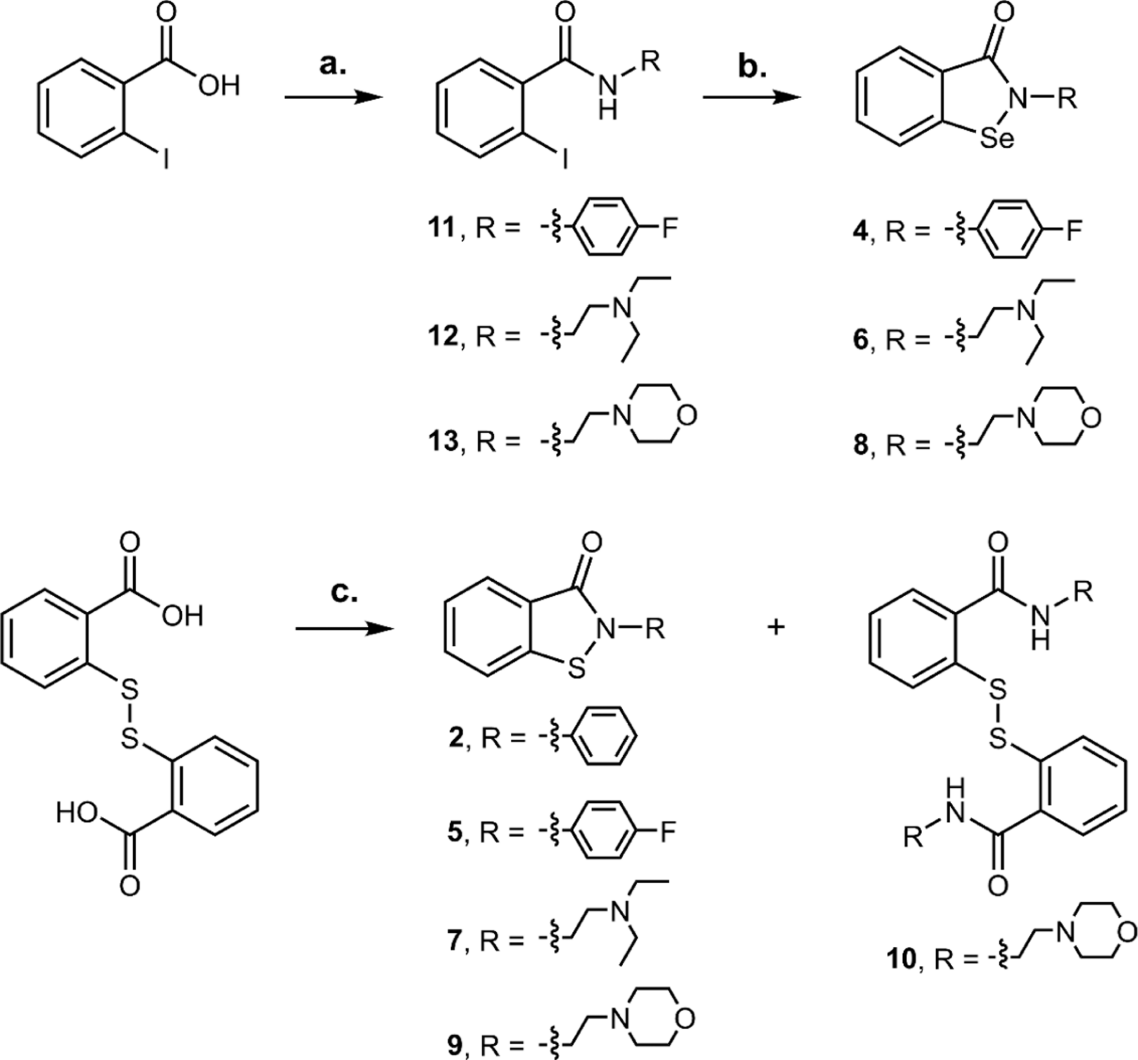
Synthesis of Se and S Analogues Reagents and conditions: (a) (i) SOCl_2_, N_2_, reflux, 3 h, (ii) amine, triethylamine (TEA), dry tetrahydrofuran (THF), N_2_, 0 °C to room temperature (RT), 16 h. (b) CuI, 1,10-phenanthroline, selenium, K_2_CO_3_, dry dimethylformamide (DMF), N_2_, 110 °C, 16 h. (c) (i) SOCl_2_, N_2_, reflux, 16 h, (ii) amine, TEA, dry THF, N_2_, 0 °C to RT, 16 h.

We introduced a fluorine atom in the para position on the phenyl amide unit (compounds **4** and **5**) as the first modification. The replacement of a hydrogen atom with a fluorine one has a prominent role as bioisostere in drug design due to its small size and high electronegativity.^48^ Furthermore, for increasing the conformational flexibility and, at the same time, increasing water solubility, we introduced a C2-alkyl chain ending with a protonable tertiary nitrogen (compounds **6** and **7**) or a morpholine ring, bearing the lone pairs on oxygen able to accept hydrogen bond (compounds **8** and **9**). The selenium compounds were synthesized starting from the appropriate 2-iodobenzamides by a copper(I)-catalyzed procedure,^49^ while sulfured analogues from commercially available 2,2-dithiobenzoic acid after treatment with thionyl chloride followed by addition of proper amine (Scheme 1). The latter reaction produces in a consistent amount also the oxidized form of dithiol (*i.e.*, compound **10**).

We investigated the capability of the ebselen-like analogues to interact with the YTHDF1 YTH domain using the tryptophan quenching assay (Table 1). The compounds tested, except compound **3**, behaved similarly to the lead scaffold, showing a micromolar interaction with the domain. Both the isoselenazolinone and isothiazolinone groups can form a Se–S or S–S covalent bond with the free thiol group of cysteine. Interestingly, compound **10** interacts similarly to its reduced form compound **9**. This is probably due to the nucleophilic attack of the thiol to the cysteine in disulfide bond favored by the excellent property as a leaving group of thiolate ions. YTH co-crystal structures were determined in complexes with compounds **7**, **9**, and **10**. Compound **9** establishes the disulfide bridge with Cys412, but the thiophenyl group is oriented in the opposite direction with respect to the ebselen selenophenol (Figure 7B). The thiophenyl moiety inserts into the groove defined by Asp401, Arg404, Ser405, Ile410, and Trp411, instead occupied by the *N*-phenyl moiety in the ebselen complex, by Trp470 in the apo YTH, and by Trp465 in the RNA-bound structure (Figure 7C). Interestingly, Trp465 is called back by the thiophenol group that is now sandwiched between Trp411 and Trp465. The diethylamine tail points toward the solvent forming a salt bridge with Asp401, which is also in hydrogen-bond contact with the central amide nitrogen.

**Table 1:**
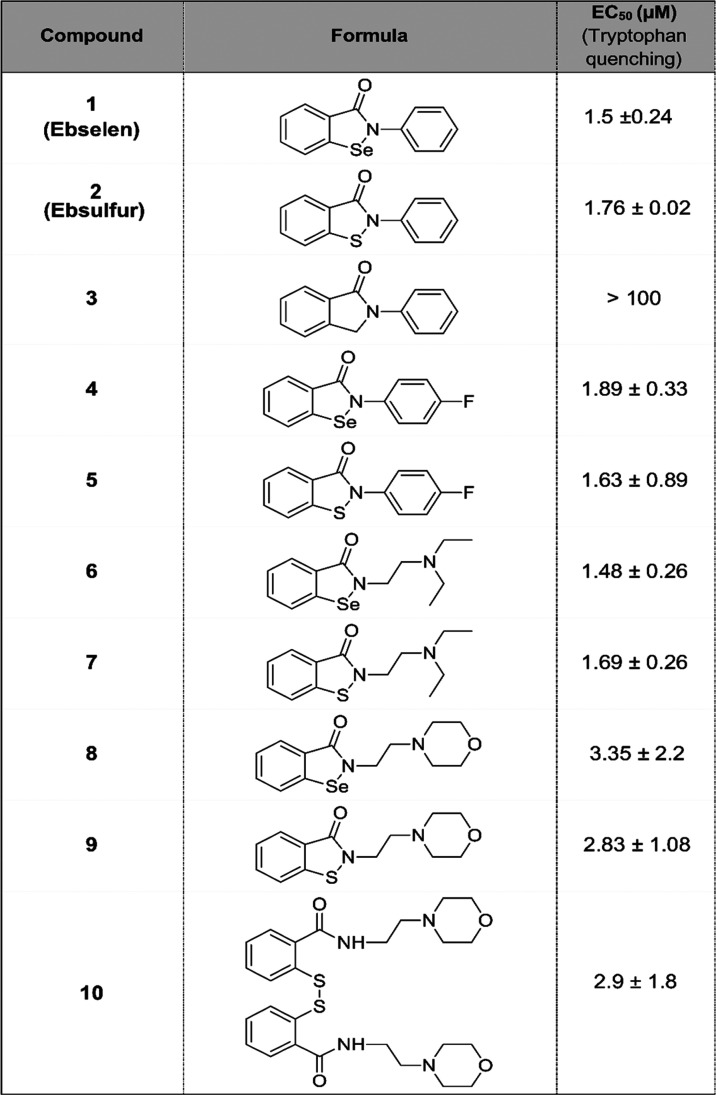
Ebselen-like Analogues Interact with the YTH Domain^a^

a All compounds show similar EC_50_.

The complexes with compounds **9** and **10** are identical as the disulfide in compound **10** is reduced by Cys412 returning compound **9**. The thiophenyl is again displaced with respect to ebselen and located similarly to compound **7** (Figure 7C). Instead, the morpholine ring assumes an opposite orientation, being directed toward the m6A site and occupying the position of Trp470 in the RNA-bound structure (Figure 7D); it establishes van der Waals interactions with Tyr397 and Thr414.

In the YTH crystallographic structures presented here, either in complex with ebselen or its analogs, an aromatic ring occupies the small pocket defined by Asp401, Arg404, Ser405, Ile410, and Trp411, which then configures as the major hotspot. Binding poses of ebselen, compounds **7** and **9** diverge in the remaining regions and provide interesting growing vectors either toward the m6A pocket or residues lining it, as valuable determinants for increasing target specificity.

## Discussion

Here, we describe the successful synthesis of the first-in-class series of small molecules that bind to the YTH domain of the YTHDF proteins. We identified the small-molecule ebselen by proof-of-principle high-throughput screening as a disruptor of the interaction between the YTH domain RNA in the micromolar range, by being a direct binder of the protein. Ebselen is a synthetic organoselenium small molecule proposed to have at least two mechanisms of action in cells. From one side, it can mimic the activity of glutathione peroxidase (GPx) *in vitro* and *in vivo*(34,35) and act as an antioxidant agent with cytoprotective properties. From the other side, it can form, as in our case, selenenyl–sulfide bonds with the thiols of cysteinyl residues. This mechanism is indicated as responsible for the majority of the biological effects of ebselen, as it can target cysteines affecting the activities of the different proteins. The same antioxidant effect is now mainly ascribed to the oxidation of cysteinyl residues in the Keap1 protein, which activates the Nrf2 signaling pathway and the subsequent transcription of antioxidant enzymes,^32,50^ more than to the mimicking of the GPx biochemical activity. The pharmacokinetic profile of ebselen indicates that it is transported in the blood by serum albumin *via* a covalent Se–S bond but can be exchanged with other proteins and transferred within cells according to the presence of glutathione and reactive thiol groups and assuming fast equilibria between all thiol-containing constituents of the system. However, the binding pocket of specific proteins can provide additional weaker forces driving target binding selectivity.^51,52^

Ebselen has been preclinically evaluated in phase 2 clinical trials for mania and hypomania in bipolar, diabetes, and antimicrobial activity, with positive results.^52−55^ Currently, it is under investigation in clinical trials for sensorineural hearing loss^32,33^ and COronaVIrus Disease 19 (COVID-19). Notably, ebselen has been recently identified as a covalent binder of the papain-like protease (PL^pro^)^44^ as well as of the main protease (M^pro^)^45^ of the SARS-CoV-2 virus. These observations clearly point at various mechanisms of action of ebselen within different cellular contexts.

The three human YTHDF protein paralogs are homologous in sequence and predicted plasticity.^56^ The generation of a selective inhibitor configures a complicated task; however, the absolute need to discriminate between the paralogs is to be established, given their reported overlapping functions.^16,57^ Specificity against the nuclear YTHDC proteins seems instead close at hand considering the more significant sequence divergence and the substitution of YTHDF1 Cys412 (conserved in YTHDF2 and YTHDF3) with a serine residue in both YTHDC1 and YTHDC2. In addition, the YTHDC proteins appear to have a clear, distinct localization and function from the YTHDF ones. Here, we have shown that ebselen interacts with the YTH domains of YTHDF1 and YTHDF2 and, in the cellular context, disrupts the interaction with target mRNAs. The fact that ebselen interacts either covalently or reversibly with the YTH domains opens the perspective of exploiting its chemical structure for a medicinal chemistry effort and producing more selective and potent analogues. X-ray and NMR studies demonstrate that the region of the ligand–protein interaction is close to the binding pocket. Although the covalent and noncovalent binding modes appear not to be perfectly superimposable, both clearly interfere with the YTH binding pocket for methylated RNAs.

Ebselen-like compounds have already been synthesized.^58^ In the case of SARS-CoV-2 M^pro^, crystallographic and mass spectrometry data suggest that only Se is retained in the catalytic site of the enzyme caused by the hydrolysis of the enzyme-bound, organoselenium covalent adduct and formation of a phenolic byproduct.^45^ However, preliminary structural–activity relationships based on enzymatic inhibition of *Bacillus anthracis* thioredoxin reductase and *Bacillus subtilis* bacterial growth have been described, suggesting the relevance of substituents in determining the pharmacological activity.^59^ More importantly, after the identification of ebselen as an inhibitor of *Mycobacterium tuberculosis* (Mtb) antigen 85 complexes (Ag85C),^60,61^ two analogous were co-crystallized in the presence of Mtb Ag85C, displaying covalent modification of the noncatalytic Cys209 residue, forming a selenenyl–sulfide bond with the derivatives.^62^ Similarly, in our case, ebselen analogues bind covalently to the YTH domain. Interestingly, analogues **7** and **9** occupy the same specific small pocket of ebselen but the different substituents affect the binding poses of both the selenophenyl/thiophenyl headgroups and the tail moieties. This evidence suggests that our chemical class of analogs can evolve toward a more potent and specific lead compound than ebselen for *in vivo* applications.

Notably, ebselen could be used as a molecular probe to study the mechanisms of the m6A signal transduction and how its alteration contributes to disease. Immediate use in genome-wide experiments is aimed at elucidating how the inhibition of the YTHDF proteins can modulate the fate of target RNAs and impact cell physiology. In summarizing, we show here, for the first time to the best of our knowledge, a small molecule endowed with the property of inhibiting the binding to methylated RNA of the YTH domain of the YTHDF proteins. We also report the synthesis of a small number of ebselen-like analogues that recapitulate the binding properties of ebselen to the YTH domain. Therefore, we have demonstrated that drug-like small molecules can interfere with the specific reading ability of the YTHDF RNA-binding proteins, opening the druggability of this class of proteins. We envision that the refinement of the reported analogues or other small molecules can lead to identifying a specific and potent binder of YTHDF proteins, acting as a pharmacological modulator of m6A.

## Materials and Methods

### Expression and Purification of the Recombinant YTH Domain of YTHDF1 and YTHDF2

The human YTHDF1 YTH domain (amino acids 365–554, PDB: 4RCJ), (Addgene plasmid # 64654, plasmid pET28a-MHL, 6× His Tag at the C-terminal), and the human YTHDF2 YTH domain (amino acids 383–553, PDB: 4WQN, cloned in pET21b(+) 6× His Tag at the N-terminal) were expressed in the BL21(DE3) *Escherichia coli* strain cultured in a Luria–Bertani medium at 37 °C till OD = 0.6–0.8 and then shifted at 18 °C O/N after induction with 0.5 mM isopropyl-β-d-thiogalactopyranoside (IPTG). After centrifugation (8500*g* for 30 min at 4 °C), bacterial pellets were lysed by sonication (six alternate pulses of 30 s, in ice) in 20 mM *N*-(2-hydroxyethyl)piperazine-*N*′-ethanesulfonic acid (Hepes) (pH 7.5), 300 mM NaCl, and with protease inhibitors. Precipitates were removed by centrifugation (15,000 rpm for 30 min at 4 °C). The protein was purified using a Ni-chelating resin (Quiagen) after imidazole elution (20, 50, 300 mM) and dialyzed O/N with 20 mM Hepes (pH 7.5), 150 mM NaCl, and 5 mM 2-mercaptoethanol. The recombinant proteins were analyzed with Coomassie blue staining after sodium dodecyl sulfate-polyacrylamide gel electrophoresis (SDS-PAGE), and the concentration was determined with both the Bradford assay method and through the extinction coefficient calculation (*C* = *A*/ε × *L*, *L* = 1 cm).

### Protein Purification for X-ray Crystallography, Crystallization, and Structure Solution

Mutations E544A/E545V/E546V were introduced in the pET28-MHL_YTH 4RCI plasmid (Addgene #64653) using back-to-back mutagenesis. The crystallization-prone mutant was expressed in *E. coli* BL21 (DE3) cells. Cells were grown at 37 °C in a Luria–Bertani medium from overnight cultures and induced with 0.5 mM IPTG when the OD_600_ of the culture reached 0.6–0.8. The induction was carried out overnight at 18 °C. Cultures were harvested by centrifugation (8500*g* for 30 min at 4 °C) on a Beckman Coulter Avanti J-20 XP centrifuge and then resuspended in lysis buffer (20 mM Hepes, pH 7.5 at 20 °C, 200 mM NaCl, and 0.4 mM tris(2-carboxyethyl)-phosphine (TCEP)) in the presence of cOmplete, Mini, ethylenediaminetetraacetic acid (EDTA)-free Protease Inhibitor Cocktail (Roche) and 10 μg/mL DNAse (Sigma-Aldrich) together with 20 mM MgCl_2_. Cells were disrupted with a tip sonicator (Branson Sonifier 450) while kept on ice, and the lysate was cleared by centrifugation (16,000*g* for 40 min at 4 °C, JA 25.50 rotor, on a Beckman Coulter Avanti J-20 XP centrifuge). The 6× His-tagged protein was purified by IMAC (nickel nitrilotriacetic acid (Ni-NTA) resin) eluting with a linear imidazole gradient from 20 to 500 mM. Buffer was exchanged to 20 mM Hepes, pH 7.5, 200 mM NaCl, 0.4 mM TCEP, and 0.5 mM EDTA, and the 6× His Tag was removed with the tobacco etch virus (TEV) protease. The protein was further purified by a second IMAC and an size exclusion chromatography (SEC) using a Superdex 75 column and 20 mM Hepes, pH 7.5 at 20 °C, 0.2 M NaCl; 0.4 mM TCEP as the mobile phase. The protein was finally concentrated at 19 mg/mL and frozen in liquid nitrogen.

Crystals of YTHDF1 in complex with ebselen and its analogues were obtained by sitting drop vapor diffusion at 4 °C. The protein solution was mixed with an equal quantity of crystallization buffer with the following composition: 100 mM glycine–NaOH, pH 9.5, 200 mM KSCN, 1 mM ebselen, 6% PEG3350, and 2% DMSO. The co-crystals were then cryo-protected with a solution of identical composition as the crystallization buffer except for 21% PEG3350 and 25% ethylene glycol and frozen in liquid nitrogen.

Data were collected at the Elettra synchrotron (Trieste, Italy), XRD2 beamline, and processed as described elsewhere.^63^ Briefly, XDS and AIMLESS were used for data integration, reduction, and scaling, while a molecular replacement was performed with PHASER using PDB 4RCI as a search model. The structure was refined through alternating cycles of manual and automatic rebuilding with COOT and PHENIX, respectively. Data collection and refinement statistics are reported in the Table S3.

### NMR Assignment of YTHDF1

The NMR assignment of the YTH domain was obtained from the analysis of standard ^1^H-detected triple resonance NMR spectra [3D HNCA, 3D CBCA(CO)NH, 3D HNCACB, 3D HNCO, and 3D HN(CA)CO] acquired on a sample of the [U ^13^C-^15^N]-enriched protein (at the concentration of ∼0.5 mM, in 20 mM Tris buffer, pH 7.5, 150 mM NaCl, 250 mM LiCl, 10 mM 2-mercaptoethanol, 0.5 mM EDTA, 0.1% NaN_3_, and protease inhibitors) using a Bruker NMR DRX spectrometer operating at 500 MHz, ^1^H Larmor frequency, equipped with a triple resonance cryoprobe, at 298 K. The assignment was helped also by the analysis of 2D ^13^C-detected experiments (2D CBCACO, 2D CACO, and 2D CON) acquired on a Bruker Avance NMR spectrometer operating at 700 MHz, ^1^H Larmor frequency, equipped with a cryogenically cooled probe optimized for ^13^C sensitivity (TCI, S/N 1500:1, on the ASTM standard sample). The large signal overlap in the 2D ^1^H–^15^N HSQC and the loss of the signals in the 3D NMR experiments, because of unfavorable relaxation phenomena, possibly due to protein aggregation, prevented the complete resonance assignment. Only 70% of the protein sequence could be assigned. NMR assignment could be, then, extended up to 80% with the help of a perdeuterated sample [U-2H-^13^C-^15^N] and the acquisition of 3D NMR spectra with the TROSY scheme^64^ [tr-HNCA and tr-HNCACB] on a Bruker AvanceIIIHD NMR spectrometer operating at 950 MHz, ^1^H Larmor frequency, equipped with a triple resonance cryoprobe. An additional 3D tr-HNCA spectrum was acquired on the perdeuterated protein in a buffer at a lower pH value (20 mM Tris buffer at pH 6.8, 150 mM NaCl, 250 mM LiCl, 10 mM 2-mercaptoethanol, 0.5 mM EDTA, 0.1% NaN_3_, and protease inhibitors). All of the spectra were processed with the Bruker TopSpin 3.6 software package and analyzed with the program CARA.

### Interaction of YTHDF1 with Ebselen and m6A RNA Monitored by NMR

The interaction of the protein with ebselen was investigated by solution NMR. 2D ^1^H–^15^N HSQC spectra were acquired on a spectrometer operating at 950 MHz and 298 K, on the free protein (100 μM in 20 mM Tris buffer, pH 7.5, 150 mM NaCl, 250 mM LiCl, 10 mM 2-mercaptoethanol, 0.5 mM EDTA, 0.1% NaN_3_, and protease inhibitors) and after the addition of increasing aliquots of a solution of the ligand in DMSO-*d*_6_ to reach the final concentrations of 12.5, 25, 50, 100, and 200 μM. The binding region of the methylated RNA segment [CCGGm6ACUGUC, later on m6A–RNA] on the YTHDF1 protein has been investigated by monitoring the effects in the 2D ^1^H-^15^N HSQC solution NMR spectrum of the ^15^N isotopically enriched protein upon the addition of increasing amounts of the RNA fragment. The spectra were acquired on a Bruker Avance 950 MHz NMR spectrometer at 298 K on a buffered solution [20 mM Tris, pH 7.5, 150 mM NaCl, 250 mM LiCl, 80 mM KCl, 10 mM β-ME, 0.5 mM EDTA, 0.1% NaN_3_, and protease inhibitors] of the protein at the concentration of 100 μM. Increasing amounts of the m6A–RNA fragment [10, 20, 40, 100, and 150 μM] were added to the protein solution during the NMR titration.

### Fluorescence Spectroscopy and Binding Assay

Black 384-well plates were filled with 5 μM protein, and the protein was incubated with increasing concentrations of adenosine (Sigma-Aldrich), *N*^6^-methyladenosine (Selleckchem), or two oligoribonucleotides containing two variants of the m6A consensus sequence, methylated or unmethylated in A (5′-CCGGm6ACUGUC-3′/5′-CCGGACUGUC-3′;5-′CCGAm6ACUGUC-3′/5′CCGAACUGUC-3, Dharmacon), in 20 mM Hepes (pH 7.5), 150 mM NaCl, and 10% glycerol. Fluorescence was measured using a Tecan Infinite 200 Microplate reader (Tecan Group Ltd.), setting the emission wavelength at 288 nm and collecting the emission data at 330 nm.

### High-Throughput Screen

The *Z* factor of the fluorescence quenching assay was calculated by incubating 1 mM *N*^6^-methyladenosine with the protein (5 μM) in 16 wells and measuring its quenching effect upon binding with the formula: *Z* = 1 – 3(σ_p_ + σ_n_)/(μ_p_ – μ_n_), where σ is the standard deviation, μ is the mean, and (p) and (n) are the positive and negative controls, respectively. A *Z* factor of 0.53 was obtained. The high-throughput screen was performed by using an automatic liquid handling (Freedom EVO, Tecan), filling black 384-well plates with 15 μL of 5 μM protein in 20 mM Tris (pH 7.5), 150 mM NaCl, and 10% glycerol, and then by adding the library of compounds at a final concentration of 10 μM. Fluorescence was measured immediately after compound addition. The library used was the Spectrum Collection (Microsource Discovery System, Inc.), composed of 2560 compounds, of whom 60% are clinically used drugs, 25% natural products, and 15% other bioactive molecules. The *Z*-score was calculated as (*X* – μ_p_)/σ_p_, where *X* is the fluorescence intensity of the protein. Candidate molecules were considered all of the ones that were under the threshold of *Z* = −3, while all of the ones over that value were discarded. The threshold was selected as it indicates that 99.99% of the compounds that induce a quenching are contained in the interval [−3:3]. Selecting a compound out of this interval as a hit, *i.e.*, an effective small molecule in reducing tryptophan quenching, implies that we are assuming the risk of choosing a false positive hit with a 0.27% probability.

### RNA Electrophoretic Mobility Shift Assay (REMSA)

Competitive REMSA was performed by incubating 500 nM protein with various concentrations of ebselen (0.1–10 μM) and 2 nM 5′-IRDYE-700 conjugated RNA probe (Metabion), 5′-CCGAm6ACUGUC-3, in 20 mM Hepes (pH 7.5), 50 mM KCl, 0,5 μg bovine serum albumin (BSA), and 0.25% glycerol, in a final volume of 20 μL. The reaction was loaded on a 6% polyacrylamide gel with 0.5% glycerol. The run was performed in a 0.5× TBE buffer at 80 V and 4 °C for 40 min and then at 100 V for 20 min. Probe fluorescence was detected with the Odyssey CLx Imaging System (Licor Biosciences) using the infrared filters.

### Dynamic Mass Redistribution

The protein was immobilized in a final volume of 15 μL/well of a 50 μg/mL solution in 20 mM sodium acetate buffer, pH 6, onto the surface of label-free microplates by amine-coupling chemistry. Different concentrations of ebselen (300 nM to 50 μM) were added to the plate and the mass of the molecular complex was measured every minute for 1 h.

### AlphaScreen Assay

The Amplified Luminescent Proximity Homogeneous Assay (ALPHA Assay) was performed in white 384-well Optiplates (PerkinElmer) in a final volume of 20 μL, and it was first optimized by titrating both the protein and the biotinylated RNA probe (5′-Bi-CCGAm6ACUGUC-3′, Dharmacon) to find the appropriate right protein/RNA ratio before the saturation of the detection signal. Both were tested in a nanomolar range, with a series of concentrations for the YTH domain of YTHDF1 (0–250 nM) incubated with different concentrations of RNA (25–100 nM) in 25 mM Hepes (pH 7.5), 100 mM NaCl, and 0.1% BSA using the AlphaScreen Histidine (Nichel Chelate) detection kit (PerkinElmer). Anti-His acceptor beads (PerkinElmer) (20 μg/mL final concentration) and streptavidin donor beads (20 μg/mL final concentration) were added and the reaction was incubated in the dark at room temperature for 1 h to reach equilibrium. Light signals were detected with an Enspire Multimode Plate reader (PerkinElmer). For the competitive assay, different concentrations of ebselen and its derivatives (0–100 μM) were mixed with 50 nM RNA and 50 nM protein, in the experimental condition of saturation binding. The IC_50_ of ebselen was determined from the nonlinear regression fits of the data.

### Electrospray Ionization Mass Spectrometry (ESI-MS) Analysis

The recombinant YTH domain of the YTHDF1 protein was subjected to buffer exchanges into 20 mM ammonium acetate (pH 6.8), using Zeba Spin Desalting Columns (7k MWCO, Thermo Fisher Scientific) to remove salts and unbound compounds. Protein solution (10 μM) was then diluted 1:1 (v/v) with 50% high-performance liquid chromatography (HPLC)-grade acetonitrile and 0.1% formic acid (v/v). Samples were introduced into the mass spectrometer using a syringe pump (500 μL, Thermo Scientific 365JLT41) at a flow rate of 5 μL/min and pumped through a metal needle. The solutions were injected directly into the mass spectrometer (Fusion, Thermo Scientific, San Jose, CA) equipped with an electrospray ionization (ESI) source. The temperature of the ion transfer tube was set at 275 °C. The analysis was performed in positive mode (spray voltage 3500 V) and mass spectra were acquired over the 700–2000 *m*/*z* range using the in-source fragmentation (SID = 50). The instrument was controlled and data were acquired using the Tune Software v3.3. Data were analyzed using the Xcalibur 4.0 (Thermo Scientific). The ion series was transformed into a single molecular mass using the Xtract algorithm.

### Cell Viability Assays

PC-3 cells were seeded and treated in 96 plates for 24, 48, and 72 h. Cell viability was assessed by adding to the cells 10% of the culture medium volume of the OZBlue reagent (OZ Biosciences). Cells were incubated at 37 °C for 1 h. Fluorescence was then determined (excitation 560 nm and emission 590 nm) by a Tecan microplate reader. Cell survival was calculated with respect to control (DMSO), and IC_50_ values were determined by fitting with GraphPad Prism software.

### RNP Immunoprecipitation Assay (RIP)

Five million cells were used for each RIP experiment followed by qRT-PCR,^65^ without cross-linking steps and using 1–15 μg of the YTHDF2 antibody (Proteintech, 24744-1-AP) or the same amount of the rabbit normal IgG isotype (negative control, Cell Signaling, 2729S). Cells were harvested after 24 h of treatment with 25 μM ebselen and DMSO as the control, lysed with 20 mM Tris–HCl at pH 7.5, 100 mM KCl, 5 mM MgCl_2_, and 0.5% NP-40 for 10 min on ice, and centrifuged at 15,000*g* for 10 min at 4 °C. Lysates were then incubated with Dynabead A/G (Thermo Fisher, 10001D/10003D) for pre-clearing for 1 h at 4 °C and then with Dynabeads A/G (80/20 ratio) at RT for 1 h with YTHDF2 or rabbit isotype IgG for Ab coating. After the pre-clearing steps and the coating, lysates were split between YTHDF2 and IgG-coated beads, while 1–5% of the lysate was stored as the input and incubated O/N at 4 °C. Finally, samples were washed with NT2 buffer for five times, 5 min each at 4 °C. TRIzol reagent was then added directly to the beads for RNA extraction following the protocol described before. After RNA extraction, samples were processed for qRT-PCR, after cDNA synthesis following the kit manufacturer’s instructions (Thermo Scientific, K1612), using Universal SYBR Master Mix (KAPA Biosystems, KR03089) on CFX 96/384 Thermal Cyclers. Ct values for YTHDF2 and IgG IP were subtracted from the Ct value of the housekeeping gene 18S to yield the ΔCt value. For each condition, the ΔCt value of IgG and YTHDF2 was evaluated in triplicate. Normalization of the values was then carried out following the percent input method, in which values from IgG and YTHDF2 IP were calculated as % input of the adjusted input values. The adjusted input corresponds to the input ΔCt subtracted from the log_2_ of the input dilution factor. YTHDF2 and IgG fractions were calculated as % input by subtracting to the adjusted input values their ΔCt values. YTHDF2 IP values were then normalized on IgG.

### Total RNA Extraction and qRT-PCR

Total RNA was extracted with TRIzol reagent followed by chloroform precipitation and by DNAse I treatment for 10 min at 37 °C. cDNA synthesis was carried out following the manufacturer’s instructions of the cDNA synthesis kit (Thermo Fisher), using 1 μg of the RNA template and an equimolar mix of random and oligo-dT primers. qRT-PCR conditions were 3 min at 95 °C, followed by 39 cycles of 15 s at 95 °C alternating with 60 °C for 15 s. Melting curve analysis was performed in every reaction to confirm the presence of a single amplicon. For RIP experiments, qRT-PCR experiments were performed in triplicates and normalized on 18S or actin internal controls according to the conditions.

### m6A Stability Measurements

PC-3 cells were plated on a six-well dish. At 70–80% confluency, cells were treated with 5 mg/mL actinomycin D or vehicle (DMSO) to inhibit transcription in combination with 50 μM ebselen for 4 h before collection. As a control, cells were treated with actinomycin D or vehicle (DMSO) for 4 h. Total RNA was isolated from cells using TRIzol according to the manufacturer’s instructions. For each condition, the same amount of total RNA was reverse-transcribed to cDNA (2 μg) using the SuperScript IV First-Strand kit. Oligo-dT primers were used during the cDNA synthesis step. This allowed us to selectively convert to cDNA the amount of intact RNA still present in cells upon actinomycin D treatment while avoiding the conversion of fragmented RNA to cDNA. qRT-PCR experiments were performed in duplicates and normalized on a stable mRNA, GAPDH.

### Thin-Layer Chromatography

Relative levels of internal m6A were determined by thin-layer chromatography (TLC), as described previously.^66^ m6A measured using TLC does not have the problem of potential contamination by ubiquitous ribosomal RNA m6A and snRNA m6A since these m6A sites are found in a consensus site that prevents its detection by TLC. 100 ng of the poly(A) purified RNA was used as the input. Processed samples were analyzed on glass-backed PEI-cellulose plates (MerckMillipore) as described previously. Plates were exposed to a storage phosphor screen below saturation and processed on a Typhoon NIR laser scanner (Cytiva). Quantification of individual nucleotides was done with ImageJ. The relative amount of m6A was calculated as a percent of the total A (sum of both A and m6A spot intensity).

### YTHDF PAR-CLIP Analysis

PAR-CLIP datasets for YTHDF1, YTHDF2, and YTHDF3 were obtained from GEO with IDs GSE63591, GSE49339, and GSE86214, respectively.^11,38,39^ Genes annotated to significant peaks were extracted and lists of target genes for each replicate were intersected to obtain a final list of consistently targeted genes for each RBP. Genes were ranked by their peak significance and compared between the three datasets to select those most consistently present in the top 10 or 100 of the ranking of more than one RBP. We selected PEG10 and NOTCH2, found in the top 10 of YTHDF3, the top 100 of YTHDF1, and in all three lists of targets.

### Cellular Thermal Stability Assay (CETSA)

HEK293T cells were seeded on 100 mm cell culture plate/s and allowed to adhere overnight. At 80% of confluency, cells were treated with 50 μM ebselen or DMSO as a control and incubated for 3 h at 37 °C in the CO_2_ incubator. Cells were centrifuged and resuspended in PBS and the protease inhibitor, at a concentration of (0.5–1) × 10^6^ cells /100 μL of suspension. Lysates were divided into 9–10 aliquots of 100 μL into 0.2 mL microcentrifuge tubes and incubated at a temperature gradient on a PCR machine for 3 min. After incubation, lysates were frozen and thawed five times in dry ice and at 27 °C for 3 min each. All tubes were then centrifuged at 4 °C for 20 min at 12,000 rpm. Supernatants were then collected and transferred to clean tubes with a loading buffer 5×, heated for 10 min at 95 °C, and detected with Western Blot. The blot was then plotted with a YTHDF1 (Proteintech, 17479-1-AP) and actin (Cell Signaling, 12620S) primary antibody and subsequently with anti-Rabbit HRP (Santa Cruz, sc-2357) and anti-mouse HRP (Thermo Fisher, 61-6520). Chemiluminescent detection was performed using Amersham ECL Prime (GE Healthcare).

### Synthesis of Selenium- and Sulfur-Containing Structural Ebselen Analogues

#### General Methods

All chemicals and reagents were purchased from Sigma-Aldrich or Alfa Aesar and used without further purification. Thin-layer chromatography (TLC) was carried out on Merck silica gel F254, using short-wave UV light as the visualizing agent and KMnO_4_ as developing agents upon heating. Column chromatography was achieved on Merck Si 40–63 μm. NMR spectra were recorded on a Bruker Avance 400 spectrometer using a 5 mm BBI probe, ^1^H NMR at 400 MHz and ^13^C NMR at 100 MHz in CDCl_3_ (relative to δ_H_ 7.27 and δ_C_ 77.0 ppm, respectively) with chemical shift values in ppm and *J* values in hertz. All compounds are >95% pure by HPLC. HPLC chromatograms were carried out using an reversed-phase (RP)-HPLC system and an Agilent 1200 high-performance liquid chromatography (HPLC) system equipped with an autosampler, a binary pump, a diode array detector (Agilent Technologies Waldbronn, Germany), and a Phenomenex Gemini 5u C18 110A column, in gradient conditions with eluent water/acetonitrile (CH_3_CN *t*_0_ 30%, *t*_8 min_ 80%, and *t*_22 min_ 80%) flow 1 mL/min (method A) or Luna 5u C18 100A column, in isocratic conditions with eluent water/methanol 1:9 flow 0.5 mL/min (method B) detection at 210, 254, 280, and 310 nm. Electrospray ionization (ESI) mass spectra were recorded using a Bruker Esquire-LC spectrometer by direct infusion of a methanol solution (source temperature 300 °C, drying gas N_2_, 4 L/min, scan range *m*/*z* 100–1000). High-resolution ESI-MS spectra were obtained by direct infusion of a methanol solution using an Orbitrap Fusion Tribrid mass spectrometer. NMR spectra (Figure S5) and HPLC chromatograms (Figure S6) can be found in the Supporting Information (SI).

#### General Procedure for the Synthesis of Se–N Heterocycles

A stirred solution of 2-iodobenzoic acids (0.33 mmol, 1 equiv) in SOCl_2_ (0.15 mL) was refluxed under a N_2_ atmosphere for 3 h and then dried under reduced pressure. The solid was dissolved in 1 mL of dry THF and added dropwise to an ice-cooled solution of amine (1 equiv) and TEA (2 equiv) in dry THF (2 mL). The solution was allowed to reach room temperature and stirred overnight. The solvent was evaporated and the solid was extracted with water and dichloromethane. The organic phases were dried over anhydrous Na_2_SO_4_ and concentrated under reduced pressure. The residues were purified by flash column chromatography on silica gel.

A solution of CuI (0.2 equiv) and 1,10-phenantroline in dry DMF (0.2 mL for 0.14 mmol of 2-iodobenzamides) was stirred under a N_2_ atmosphere for 15 min at room temperature. 2-Iodobenzamides (1 equiv), selenium powder (1.2 equiv), and K_2_CO_3_ (1.5 equiv) were added to the solution and heated at 110 °C for 16 h. The reaction was quenched by addition of 4 mL of brine and stirred for additional 3 h at room temperature. The reaction mixture was extracted with EtOAc (3×). The combined organic phases were washed with water, dried over anhydrous Na_2_SO_4_, and concentrated under reduced pressure. The residues were purified by column chromatography on silica gel (Figures S7 and S8).

##### *N*-(4-Fluorophenyl)-2-iodobenzamide (**11**)

White powder; yield = 42 mg (81%); *R*_*f*_ = 0.9 (CH_2_Cl_2_ (DCM)/MeOH 97:3); purification by column chromatography on silica gel DCM/MeOH 99:1; ^1^H NMR (400 MHz, CDCl_3_) δ: 7.92 (d, *J* = 8.0 Hz, 1H), 7.65–7.59 (m, 2H), 7.54 (dd, *J* = 7.6, 1.6 Hz, 1H), 7.45 (dt, *J* = 7.6, 0.8 Hz 1H), 7.43 (br s, 1H), 7.17 (dt, *J* = 8.0, 1.6 Hz, 1H), 7.09 (t, *J* = 8.8 Hz, 2H); ^13^C NMR (100 MHz, CDCl_3_) δ: 168.8, 161.3 (^1^*J*_CF_ = 243 Hz), 143.3, 141.4, 134.9, 132.9, 129.9, 129.7, 123.5 (^3^*J*_CF_ = 7 Hz, 2C), 117.1 (^2^*J*_CF_ = 22 Hz, 2C), 93.8; HPLC (method A): 9.361 min; ESI-MS: *m*/*z* 364 [M + Na]^+^, 340 [M – H]^−^.

##### *N*-(2-(Diethylamino)ethyl)-2-iodobenzamide (**12**)

Yellow oil; yield = 91 mg (62%); *R*_*f*_ = 0.4 (DCM/MeOH 9:1); purification by column chromatography on silica gel DCM/MeOH 97:3; ^1^H NMR (400 MHz, CDCl_3_) δ: 7.86 (d, *J* = 8.0 Hz, 1H), 7.44–7.34 (m, 2H), 7.10 (dt, *J* = 8.0, 2.7 Hz, 1H), 6.50 (br s, 1H), 3.51 (q, *J* = 5.6 Hz, 2H), 2.68 (t, *J* = 6.0 Hz, 2H), 2.57 (q, *J* =7.2 Hz, 4H), 1.03 (t, *J* = 7.2 Hz, 6H); ^13^C NMR (100 MHz, CDCl_3_) δ: 170.7, 143.9, 141.2, 132.3, 129.6, 129.5, 93.9, 52.6, 48.0 (2C), 38.7, 13.1 (2C); HPLC (method B): 5.477 min; ESI-MS: *m*/*z* 347 [M + H]^+^, 349 [M + Na]^+^.

##### 2-Iodo-*N*-(2-morpholinoethyl)benzamide (**13**)

White powder; yield = 80 mg (67%); *R*_*f*_ = 0.7 (DCM/MeOH 95:5); purification by column chromatography on silica gel DCM/MeOH 95:5; ^1^H NMR (400 MHz, CDCl_3_) δ: 7.86 (d, *J* = 7.6 Hz, 1H), 7.45–7.36 (m, 2H), 7.11 (t, *J* = 7.6 Hz, 1H), 6.40 (br s, 1H), 3.75–3.67 (m, 4H), 3.55 (dt, *J* = 5.8, 5.3, 2H), 2.60 (t, 5.8 Hz, 2H), 2.55–2.47 (m, 4H); ^13^C NMR (100 MHz, CDCl_3_) δ: 170.7, 143.8, 141.2, 132.5, 129.8, 129.6, 93.9, 68.3 (2C), 58.1, 54.7 (2C), 37.6; HPLC (method A): 5.477 min; ESI-MS: *m*/*z* 361 [M + H]^+^, 383 [M + Na]^+^.

##### 2-(4-Fluorophenyl)benzo[*d*][1,2]selenazol-3(2*H*)-one (**4**)

White powder; yield = 21 mg (49%); *R*_*f*_ = 0.3 (DCM 100%); purification by column chromatography on silica gel DCM 100%; ^1^H NMR (400 MHz, CDCl_3_) δ: 8.13 (d, *J* = 8.2 Hz, 1H), 7.68 (d, *J* = 4.0 Hz, 2H), 7.62–7.56 (m, 2H), 7.52–7.47 (m, 1H), 7.14 (t, *J* = 8.8 Hz, 2H); ^13^C NMR (100 MHz, CDCl_3_) δ: 165.9, 159.9 (^1^*J*_CF_ = 240), 137.6, 134.9, 132.6, 129.5, 127.5 (^3^*J*_CF_ = 8 Hz, 2C), 127.1, 126.7, 123.8, 116.2 (^2^*J*_CF_ = 24 Hz, 2C); HPLC (method B): 6.795 min (96.9%); ESI-MS: *m*/*z* 316 [M + Na]^+^; HR-ESI-MS: calcd for C_13_H_9_FNOSe 239.98279; found *m*/*z* 239.98424, calcd for C_13_H_8_FNOSeNa 315.96473; found *m*/*z* 315.96592.

##### 2-(2-(Diethylamino)ethyl)benzo[*d*][1,2]selenazol-3(2*H*)-one (**6**)

White powder; yield = 10 mg (18%); *R*_*f*_ = 0.5 (DCM/MeOH 9:1); purification by column chromatography on silica gel DCM/MeOH 95:5; ^1^H NMR (400 MHz, CDCl_3_) δ: 8.04 (d, *J* = 7.7 Hz, 1H), 7.59 (d, *J* = 7.8 Hz, 1H), 7.54 (dd, *J* = 7.2, 7.8 Hz, 1H), 7.38 (dd, *J* = 7.3, 7.5 Hz, 1H), 4.01–3.96 (m, 2H), 2.80–2.69 (m, 6H), 1.13 (t, *J* = 7.2, 6H); ^13^C NMR (100 MHz, CDCl_3_) δ: 168.0, 143.0, 131.3, 128.0, 127.6, 125.6, 123.4, 52.7 (2C), 45.7, 41.5 (2C), 10.8 HPLC (method B): 4.769 min (96.3%); ESI-MS: *m*/*z* 298 [M + H]^+^, 321 [M + Na]^+^; HR-ESI-MS: calcd for C_13_H_19_N_2_OSe 299.06571; found *m*/*z* 228.04734, calcd for C_13_H_18_N_2_OSe 321.04766; found *m*/*z* 321.04823.

##### 2-(2-Morpholinoethyl)benzo[*d*][1,2]selenazol-3(2*H*)-one (**8**)

White powder; yield = 16 mg (36%); *R*_*f*_ = 0.6 (DCM/MeOH 9:1); purification by column chromatography on silica gel DCM/MeOH 95:5; ^1^H NMR (400 MHz, CDCl_3_) δ: 8.05 (d, *J* = 7.7 Hz, 1H), 7.62 (d, *J* = 7.5 Hz, 1H), 7.57 (dd, *J* = 7.4, 7.5 Hz, 1H), 7.40 (dd, *J* = 7.5, 7.5 Hz, 1H), 4.06–3.99 (m, 2H), 3.92–3.83 (m, 4H), 2.73–2.59 (m, 6H); ^13^C NMR (100 MHz, CDCl_3_) δ: 167.8, 141.9, 131.6, 128.2, 127.5, 125.8, 123.4, 66.9 (2C), 57.5, 53.4 (2C), 40.4 HPLC (method A): 6.148 min (97.3%); ESI-MS: *m*/*z* 313 [M + H]^+^, 335 [M + Na]^+^; HR-ESI-MS: calcd for C_13_H_17_N_2_O_2_Se 313.04498; found *m*/*z* 313.04607, calcd for C_13_H_16_N_2_O_2_SeNa 335.02692; found *m*/*z* 335.02752.

#### General Procedure for the Synthesis of S–N Heterocycles

A stirred solution of 2,2′-dithiobenzoic acid (100 mg, 1 equiv) in SOCl_2_ (0.125 mL) was refluxed under a N_2_ atmosphere for 16 h and then dried under reduced pressure. The solid was dissolved in 1 mL of dry THF and added dropwise to an ice-cooled solution of amine (2 equiv) and TEA (2 equiv) in dry THF (2 mL). The solution was allowed to reach room temperature and stirred overnight. The solvent was evaporated and the crude was extracted with water and dichloromethane (3×). The combined organic phases were dried over anhydrous Na_2_SO_4_ and concentrated under reduced pressure. The residues were purified by column chromatography on silica gel (Figures S7 and S8).

##### 2-Phenylbenzo[*d*]isothiazol-3(2*H*)-one (**2**)

White powder; yield = 25 mg (34%); *R*_*f*_ = 0.77 (Hex/EtOAc 1:1); purification by column chromatography on silica gel Hex/EtOAc 7:3; ^1^H NMR (400 MHz, CDCl_3_) δ: 8.11 (d, *J* = 8.0 Hz, 1H), 7.71 (d, *J* = 8.0 Hz, 2H), 7.66 (d, *J* = 6.0 Hz, 1H), 7.59 (d, *J* = 6.0 Hz, 1H), 7.51–7.41 (m, 3H), 7.36–7.29 (m, 1H); ^13^C NMR (100 MHz, CDCl_3_) δ: 164.1, 139.9, 137.3, 132.4, 129.4 (2C), 127.2, 127.1, 125.8, 124.9 (2C), 124.6, 120.1; HPLC (method A): 9.134 min (95.4%); ESI-MS: *m*/*z* 228 [M + H]^+^, 250 [M + Na]^+^; HR-ESI-MS: calcd for C_13_H_10_NOS 228.04776; found *m*/*z* 228.04734. calcd for C_13_H_9_NOSNa 250.02971; found *m*/*z* 250.02917.

##### 2-(4-Fluorophenyl)benzo[*d*]isothiazol-3(2*H*)-one (**5**)

White powder; yield = 19 mg (44%); *R*_*f*_ = 0.7 (Hex/EtOAc 1:1); purification by column chromatography on silica gel Hex/EtOAc 6:4; ^1^H NMR (400 MHz, CDCl_3_) δ: 8.11 (d, *J* = 7.7 Hz, 1H), 7.73–7.63 (m, 3H), 7.59 (d, *J* = 8.3 Hz, 1H), 7.47 (t, *J* = 7.3 Hz, 1H), 7.18 (t, *J* = 8.4 Hz, 2H). ^13^C NMR (100 MHz, CDCl_3_) δ: 165.6, 162.6 (^1^*J*_CF_ = 245 Hz), 141.3, 134.5, 133.9, 128.6, 128.2 (^3^*J*_CF_ = 8 Hz, 2C), 127.3, 125.9, 121.5, 117.6 (^2^*J*_CF_ = 22 Hz, 2C). HPLC (method B): 7.266 min (97.6%); ESI-MS: *m*/*z* 246 [M + H]^+^, 268 [M + Na]^+^; HR-ESI-MS: calcd for C_13_H_9_FNOSe 246.03834; found *m*/*z* 246.03974, calcd for C_13_H_8_FNOSeNa 268.02029; found *m*/*z* 268.02182.

##### 2-(2-(Diethylamino)ethyl)benzo[*d*]isothiazol-3(2*H*)-one (**7**)

Pale yellow oil; yield = 21 mg (25%); *R*_*f*_ = 0.63 (DCM/MeOH 9:1); purification by column chromatography on silica gel DCM/MeOH 9:1; ^1^H NMR (400 MHz, CDCl_3_) δ: 8.02 (dd, *J* = 8.0 Hz, *J* = 0.8 Hz, 1H), 7.58 (dt, *J* = 7.6 Hz, *J* = 1.2 Hz, 1H), 7.54 (dt, *J* = 7.6 Hz, *J* = 0.8 Hz, 1H), 7.37 (dt, *J* = 8.0 Hz, *J* = 1.2 Hz, 1H), 3.97 (t, *J* = 7.6 Hz, 2H), 2.77 (t, *J* = 7.6 Hz, 2H), 2.62 (q, *J* = 7.2 Hz, 4H), 1.06 (t, *J* = 7.2 Hz, 6H); ^13^C NMR (100 MHz, CDCl_3_) δ: 165.6, 141.7, 131.5, 126.4, 125.1, 124.5, 120.1, 52.2, 47.0 (2C), 42.3, 11.7 (2C); HPLC (method B): 3.158 min (95.7%); ESI-MS: *m*/*z* 251 [M + H]^+^, 273 [M + Na]^+^; HR-ESI-MS: calcd for C_13_H_19_N_2_OS 251.12126; found *m*/*z* 251.12073.

##### 2-(2-Morpholinoethyl)benzo[*d*]isothiazol-3(2*H*)-one (**9**)

Pale yellow oil; yield = 19 mg (22%); *R*_*f*_ = 0.48 (DCM/MeOH 9:1); purification by column chromatography on silica gel DCM/MeOH 9:1; ^1^H NMR (400 MHz, CDCl_3_) δ: 8.03 (d, *J* = 8.0 Hz, 1H), 7.82–7.51 (m, 2H), 7.38 (dt, *J* = 8.0 Hz, *J* = 1.2 Hz, 1H), 4.02 (t, *J* = 6.0 Hz, 2H), 3.79–3.72 (m, 4H), 2.69 (t, *J* = 6.0 Hz, 2H), 2.60–2.52 (m, 4H); ^13^C NMR (100 MHz, CDCl_3_) δ: 165.6, 141.6, 131.6, 126.5, 125.3, 124.4,120.1, 66.9 (2C), 57.4, 53.5 (2C), 40.9; HPLC (method B): 4.386 min (95.8%); ESI-MS: *m*/*z* 265 [M + H]^+^, 287 [M + Na]^+^; HR-ESI-MS: calcd for C_13_H_17_N_2_O_2_S 265.10053; found *m*/*z* 265.09999.

##### 2,2′-Disulfanediylbis(*N*-(2-morpholinoethyl)benzamide) (**10**)

White powder; yield = 98 mg (57%); *R*_*f*_ = 0.40 (DCM/MeOH 9:1); purification by column chromatography on silica gel DCM/MeOH 9:1; ^1^H NMR (400 MHz, CDCl_3_) δ: 7.76 (dd, *J* = 8.0, *J* = 0.8 Hz, 2H), 7.50 (dd, *J* = 7.6 Hz, *J* = 1.2 Hz, 2H), 7.35 (dt, *J* = 7.6 Hz, *J* = 1.6 Hz, 2H), 7.24 (dt, *J* = 7.6 Hz, *J* = 1.2 Hz, 2H), 6.80 (bt, 2H), 3.75–3.69 (m, 8H), 3.55 (q, *J* = 5.6 Hz, 4H), 2.61 (t, *J* = 6.0 Hz, 4H), 2.55–2.46 (m, 8H); ^13^C NMR (100 MHz, CDCl_3_) δ: 167.6 (2C), 137.0 (2C), 134.1 (2C), 131.2 (2C), 127.6 (2C), 127.3 (2C), 126.3 (2C), 67.0 (4C), 56.8 (2C), 53.3 (4C), 36.2 (2C); HPLC (method B): 5.156 min (96.6%); ESI-MS: *m*/*z* 531 [M + H]^+^, 553 [M + Na]^+^; HR-ESI-MS: calcd for C_34_H_35_N_4_O_4_S_2_ 531.20943; found *m*/*z* 531.21074.

## Supporting Information Available

Purification of the YTH domain of YTHDF1 and YTHDF2 proteins and calculation of the *Z*-factor for the quenching assay (Figure S1); purification of the YTH domain of YTHDC1 protein (Figure S2); determination of the EC_50_ of interaction between an m6A RNA probe with the YTH domain of YTHDF1 calculated with the DMR (Figure S3); protein level of the epitranscriptome apparatus and m6A level in PC-3 cells (Figure S4); Electron density map of for Ebselen and Cys412 side chain (Figure S5); specific interaction of a m6A containing RNA probe with the YTH domain of YTHDF1 (Figure S6); NMR spectra of ebselen analogues (Figure S7); HPLC analysis of ebselen analogues (Figure S8) (PDF)
Compounds and *Z* score (Table S1) (XLSX)
GSE63591_YTHDF1, GSE49339_YTHDF2, GSE86214_YTHDF3, and intersection(Table S2) (XLSX)
X-ray data collection (Table S3) (XLSX)

## Accession Codes

The YTHDF1/ebselen structure was deposited to the PDB with accession number 7PCU. Atomic coordinates and experimental data will be released upon article publication.

## Notes

A.Q., A.P., G.L., I.M., J.V., G.P., M.M., and D.S. declared that they filed a patent to protect ebselen and its analogues. S.R.J. is a scientific advisor and owns stock in 858 Therapeutics.
