# Supplementary material for: Small-Molecule Ebselen Binds to YTHDF Proteins Interfering with the Recognition of N6-Methyladenosine-Modified RNAs
Source: ACS Pharmacol Transl Sci. 2022 Sep 14;5(10):872–91. doi: 10.1021/acsptsci.2c00008 (PMC9578143; doi:10.1021/acsptsci.2c00008)
Supplement: Supplementary file 1 — pt2c00008_si_001.pdf [file pt2c00008_si_001.pdf]

Supplementary Information

The small molecule ebselen binds to YTHDF proteins interfering with the recognition of N6-methyladenosine modified RNAs

Mariachiara Micaelli[1], Andrea Dalle Vedove[1], Linda Cerofolini[2,3], Jacopo Vigna[4], Denise Sighel[1], Sara Zaccara[5], Isabelle Bonomo[1], Georgios Poulentzas[1], Emanuele Filiberto Rosatti[1], Giulia Cazzanelli[1], Laura Alunno[1], Romina Belli[6], Daniele Peroni[6], Erik Dassi[1], Shino Murakami[5], Samie R. Jaffrey[5], Marco Fragai[2,3], Ines Mancini[4], Graziano Lolli[1], Alessandro Quattrone[1*], Alessandro Provenzani[1*]

[1] Department of Cellular, Computational and Integrative Biology, CIBIO, University of Trento, Trento, Italy

[2] Magnetic Resonance Center (CERM) – Department of Chemistry "Ugo Schiff", University of Florence, Florence, Italy

[3] Consorzio Interuniversitario Risonanze Magnetiche di Metalloproteine (CIRMMP), Florence, Italy

[4] Department of Physics, University of Trento, Trento, Italy

[5] Department of Pharmacology, Weill Cornell Medicine, Cornell University, New York, NY 10065, USA

[6] Department of Cellular, Computational and Integrative Biology, CIBIO, Mass Spectrometry Facility, University of Trento, Trento, Italy

*co-corresponding authors

alessandro.provenzani@unitn.it

alessandro.quattrone@unitn.it

Figure S1

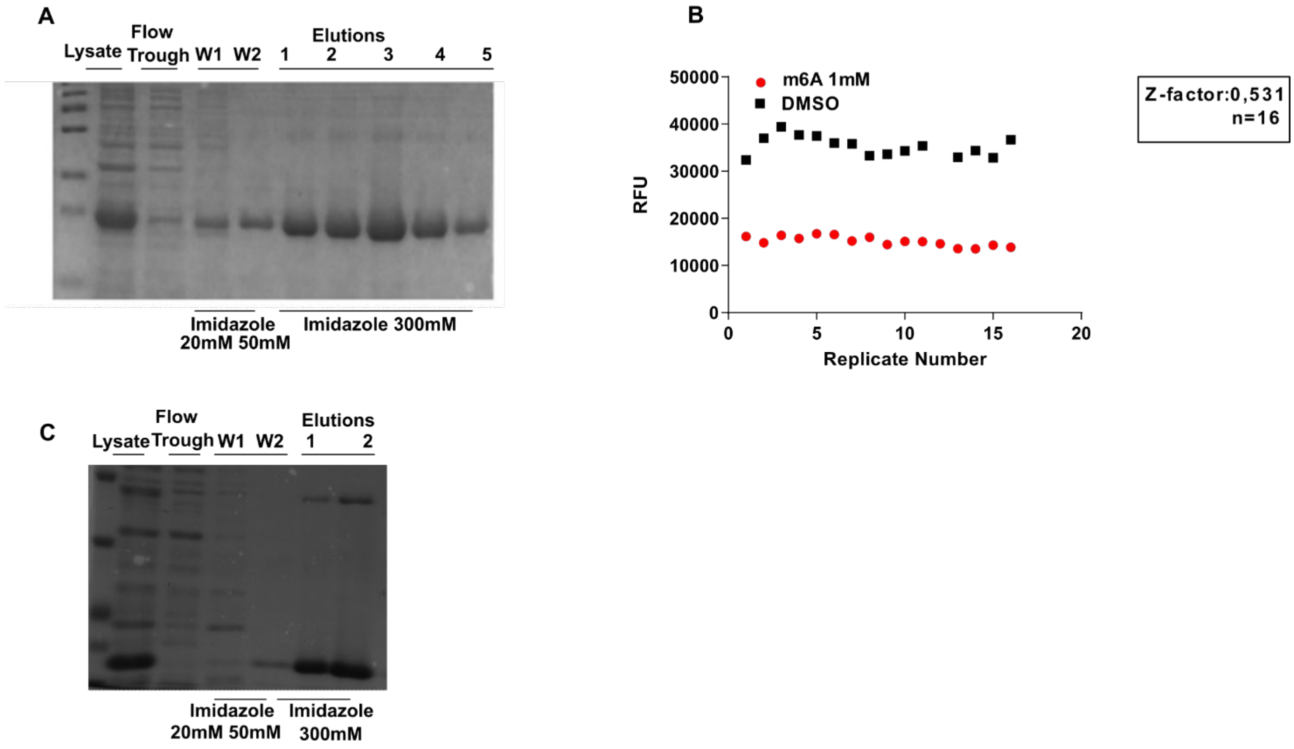

Supplementary Figure 1. (A) Purification of the YTH domain of YTHDF1 protein. SDS-PAGE analysis of purification steps of the YTHDF1 domain. Washing steps have been carried out using increasing concentrations of Imidazole (20mM-50mM). The five elution fractions have been obtained using 300mM of Imidazole*.* **(B) Calculation of the Z-factor for the quenching assay**. Quenching effect of the m6A moiety against the YTH domain has been measured in sixteen replicates, with DMSO as a negative control. Z=0.531 is an indicator of an excellent assay. **(C) Purification of the YTH domain of YTHDF2 protein.** SDS-PAGE analysis of purification steps of the YTH-DF2 domain. Washing steps have been carried out using increasing concentrations of Imidazole (20mM-50mM). The two elution fractions have been obtained using 300mM of Imidazole.

Figure S2

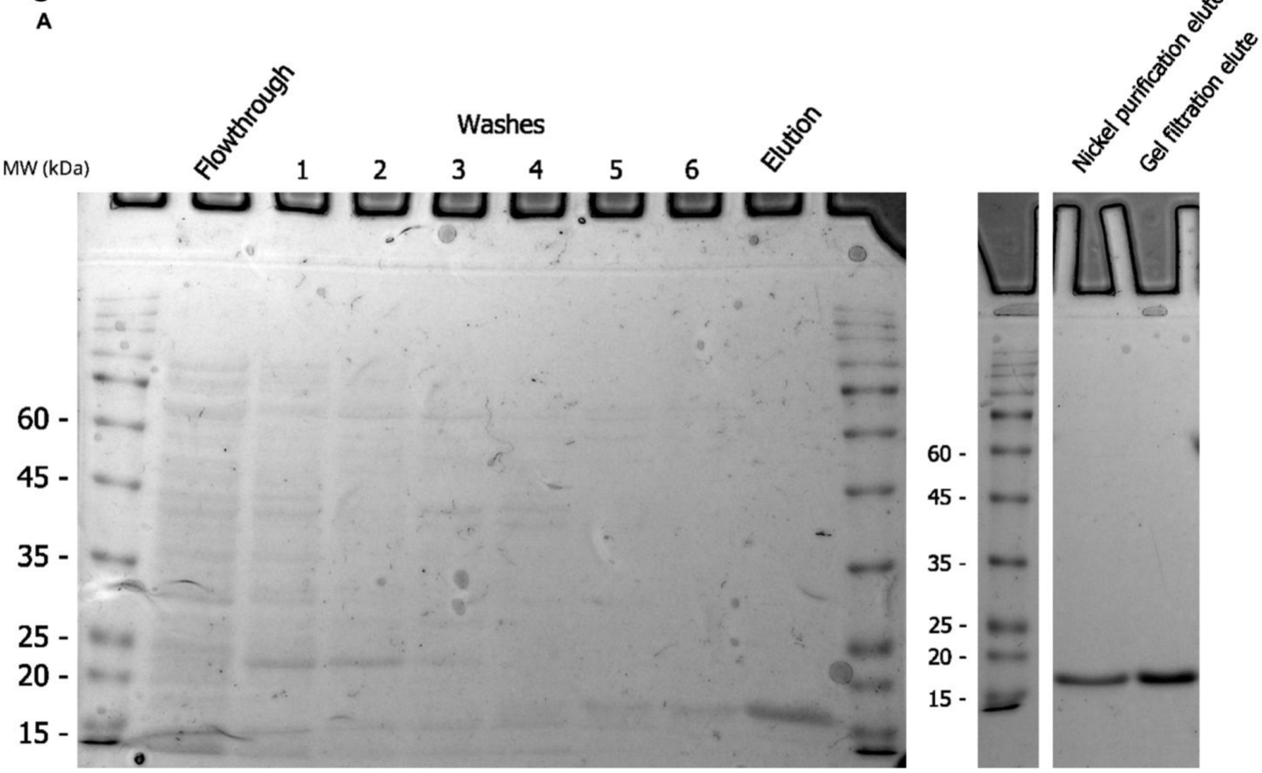

Supplementary Figure 2. Purification of the YTH domain of YTHDC1 protein. SDS-PAGE analysis of purification steps of the YTHDC1 domain with Coomassie Blue staining. Washing steps have been carried out using increasing concentrations of Imidazole (20mM-50mM). The elution fraction has been obtained using 300mM of Imidazole. Eluted protein was further purified by gel filtration.

Figure S3

A

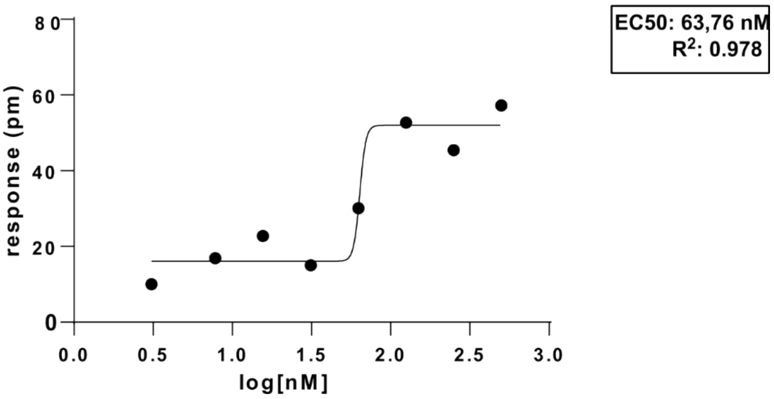

Supplementary Figure 3. Determination of the EC50 of interaction between an m6A RNA probe with the YTH domain of YTHDF1 calculated with the DMR. rYTH was immobilized to the wells with amino-coupled chemistry and different concentrations of RNA were added to the plate. Measurements were performed before adding the RNA, in order to define a baseline, and after the addition. Final response (pm) was obtained by subtracting to the last measurement the baseline. Signal for each well was obtained by subtracting the signal of a reference area with the protein coated to an uncoated one. Data were fitted according to a four-parameter nonlinear regression curve. R^2 = 0.8918 EC50 = 63.76 nM.

Figure S4

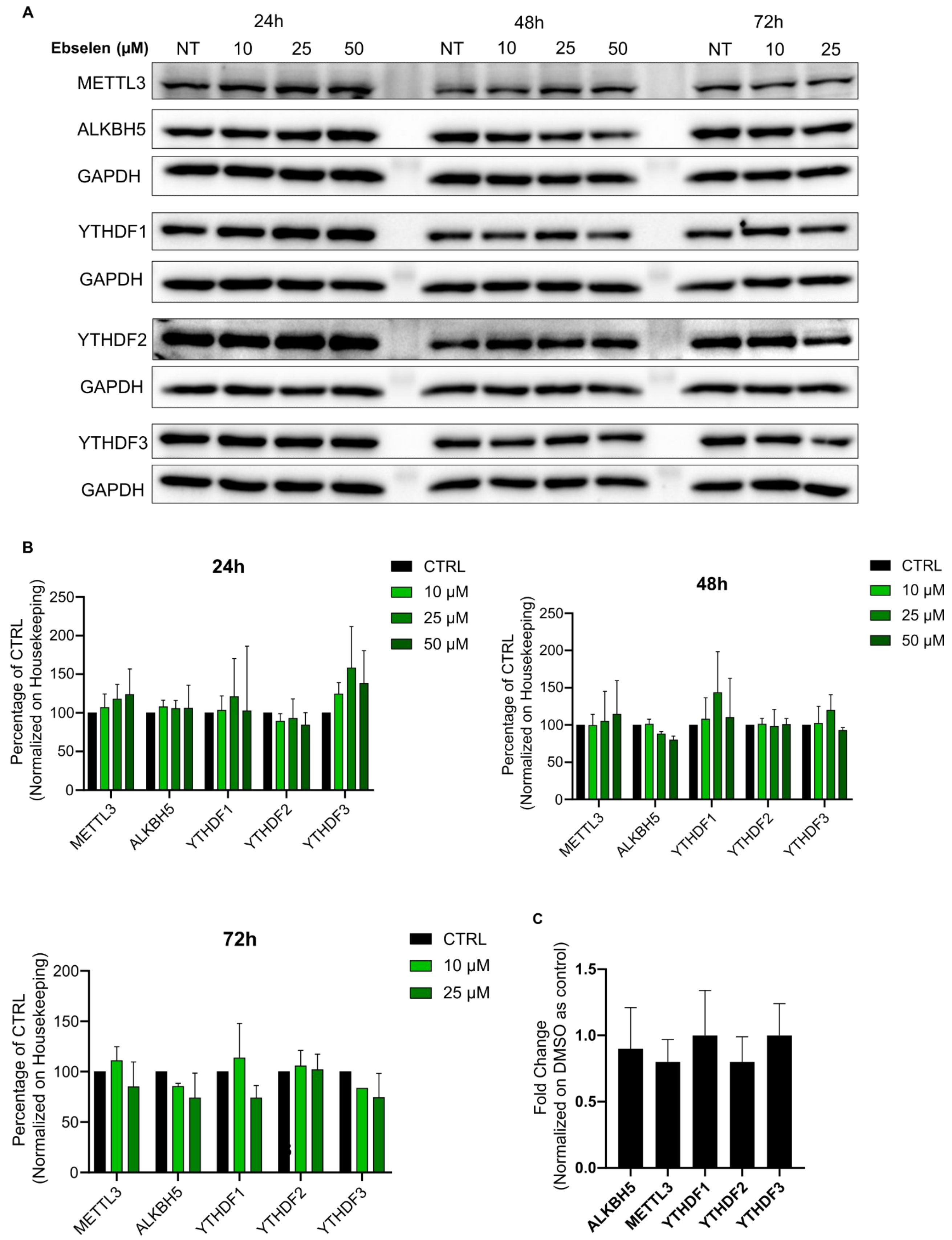
A
24h
48h
72h
Ebselen (μM)
NT
10
25
50
METTL3
ALKBH5
GAPDH
YTHDF1
YTHDF2
YTHDF3
B
24h
48h
72h
CTRL
10 μM
25 μM
50 μM
Percentage of CTRL
(Normalized on Housekeeping)
C
Fold Change
(Normalized on DMSO as control)

Supplementary Figure 4. Protein level of the epitranscriptome apparatus and m6A level in PC-3 cells. (A) Representative western blot of YTHDF1-3, ALKBH5 and METTL3 in PC-3 cells after 24, 48 and 72 hours of ebselen treatment at three doses, 10, 25, 50 μM. **(B)** Quantification of the relative changes of YTHDF1-3, ALKBH5 and METTL3 in PC-3 cells after 24, 48 and 72 hours of ebselen treatment at three doses, 10, 25, 50 μM. No changes were observed. Three biological replicates were performed. **(C)** Quantitative real-time PCR of YTHDF1-3, ALKBH5 and METTL3 in PC-3 cells after 24 hours of ebselen treatment at 10 μM.

Figure S5

A

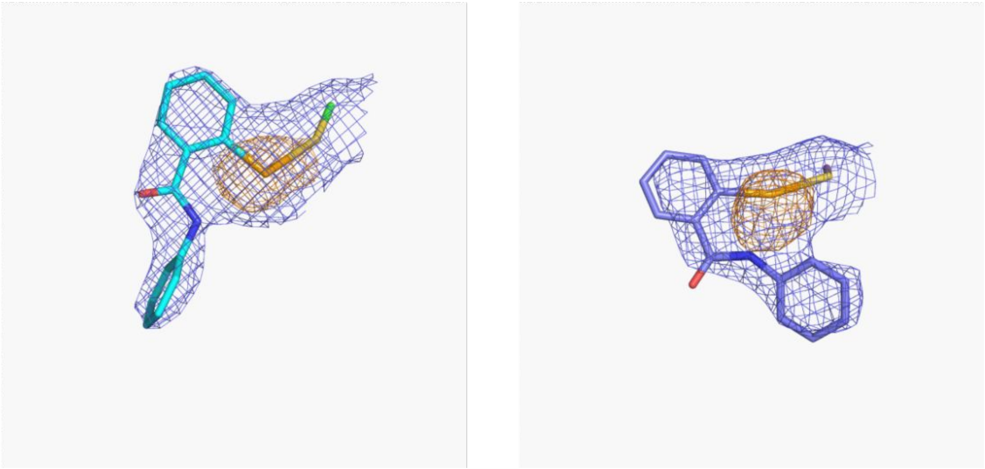

Supplementary Figure 5. Caption: $2F_o$-F_c map (blue) is contoured at 1s for Ebselen and Cys412 side chain. The selenium anomalous map is contoured at 3.5σ and shown in orange**.**

Figure S6

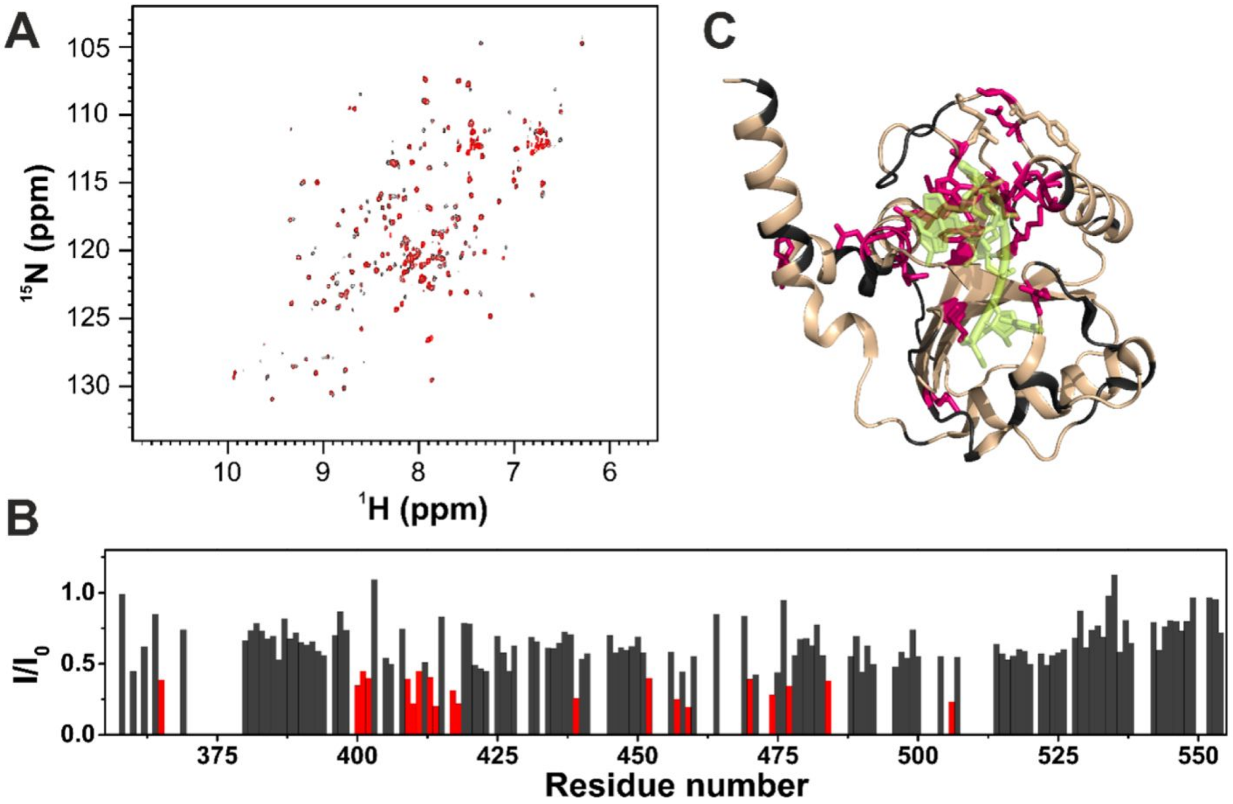

Supplementary Figure 6. Specific interaction of a m6A containing RNA probe with the YTH domain of YTHDF1. (A) Superimposition of 2D ^{1}H ^{15}N HSQC NMR spectra of free YTH domain of YTHDF1 (100 μM, black spectrum) and YTHDF1 in the presence of m6A-RNA (40 μM, red spectrum). **(B)** Intensity decreases of the signals of the YTH domain of YTHDF1 (100 μM) in the presence of m6A-RNA fragment (40 μM); the residues exhibiting the largest decreases are highlighted in red. **(C)** Cartoon representation of YTH domain (PDB code: 4rcj) in complex with a shorter RNA fragment (in yellow) with respect to the one used in the NMR titration. The residue exhibiting the largest decreases in signal intensity in the presence of m6A-RNA (40 μM) are colored in magenta and represented as sticks; in grey unassigned residues.

Figure S7 A

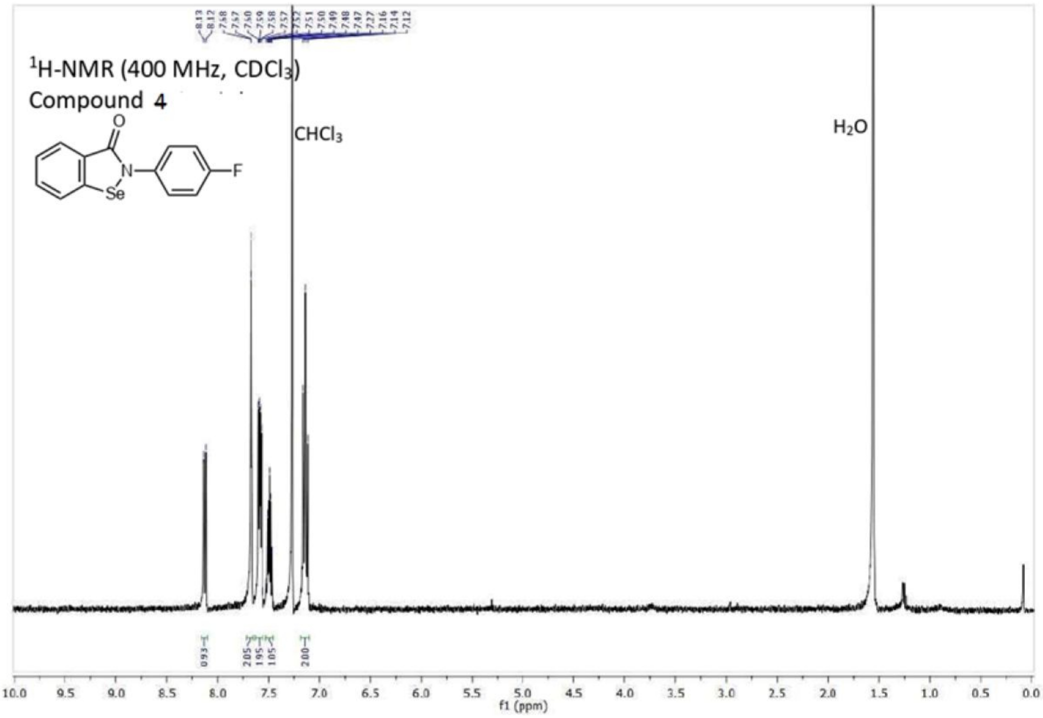

^{1}H-NMR (400 MHz, CDCl$_3$)
Compound 4
CHCl$_3$
H$_2$O
f1 (ppm)

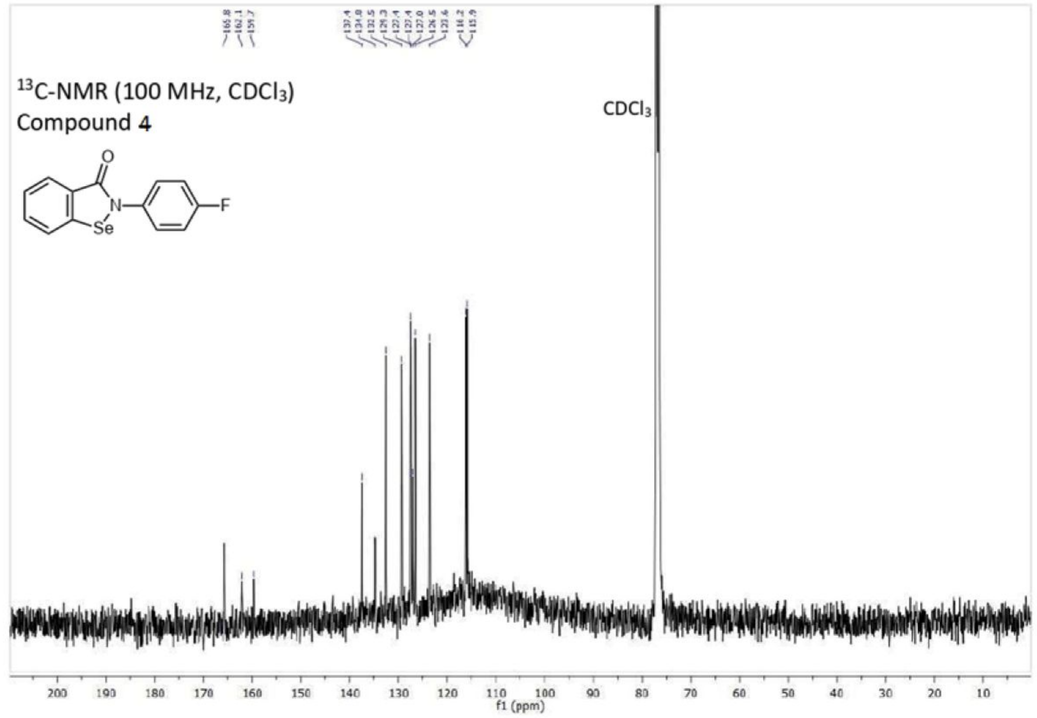

^{13}C-NMR (100 MHz, CDCl$_3$)
Compound 4
CDCl$_3$
f1 (ppm)

Figure S7 B

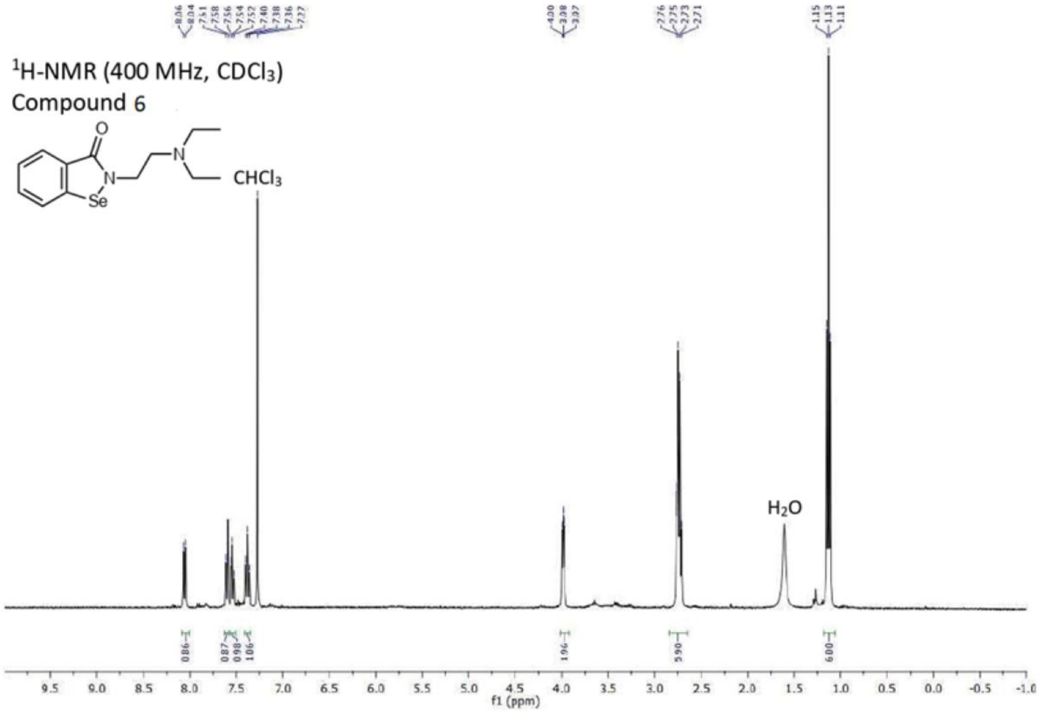

^{1}H-NMR (400 MHz, CDCl$_3$)
Compound 6
CHCl$_3$
H$_2$O
f1 (ppm)

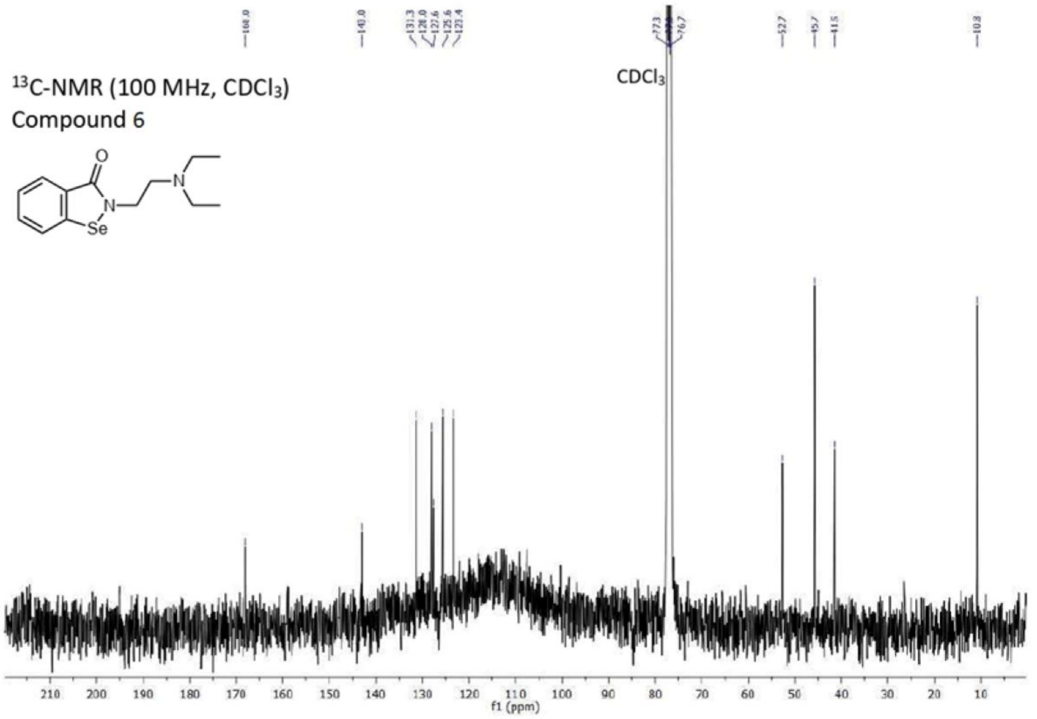

^{13}C-NMR (100 MHz, CDCl$_3$)
Compound 6
CDCl$_3$
f1 (ppm)

Figure S7 C

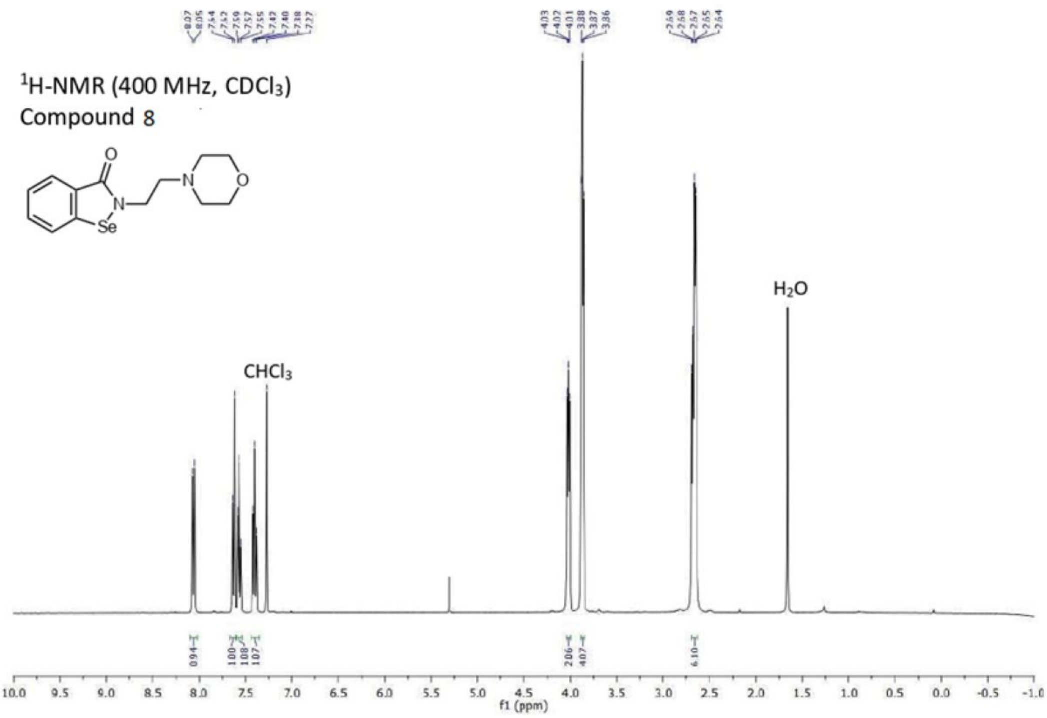

1H-NMR (400 MHz, CDCl3)
Compound 8
CHCl3
H2O
f1 (ppm)

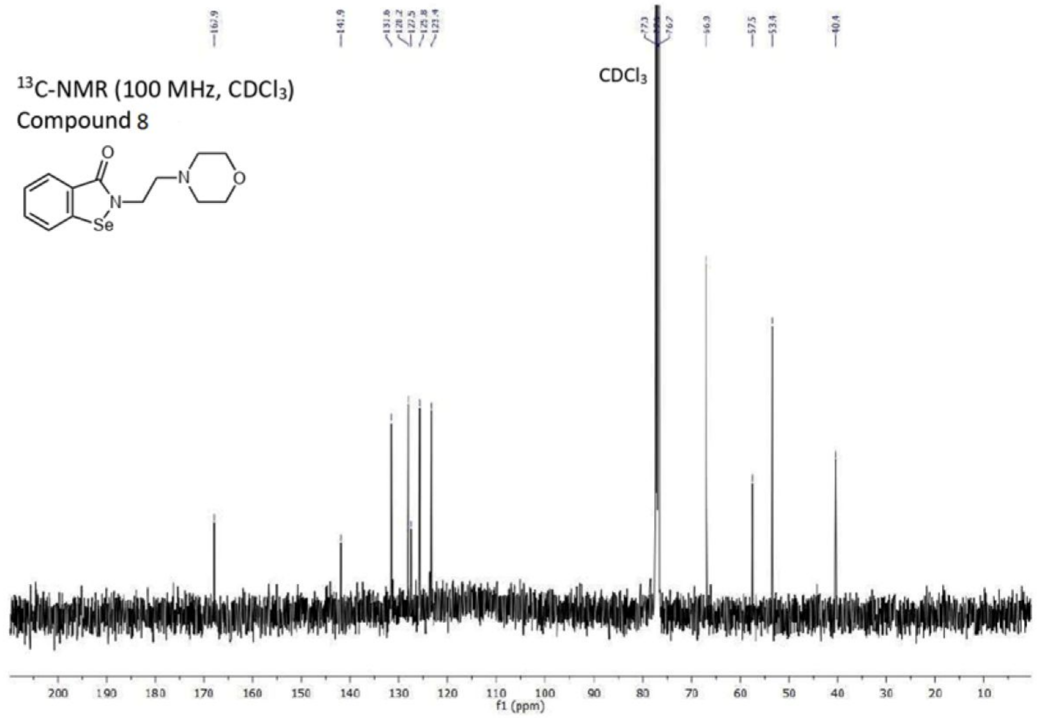

13C-NMR (100 MHz, CDCl3)
Compound 8
CDCl3
f1 (ppm)

Figure S7 D

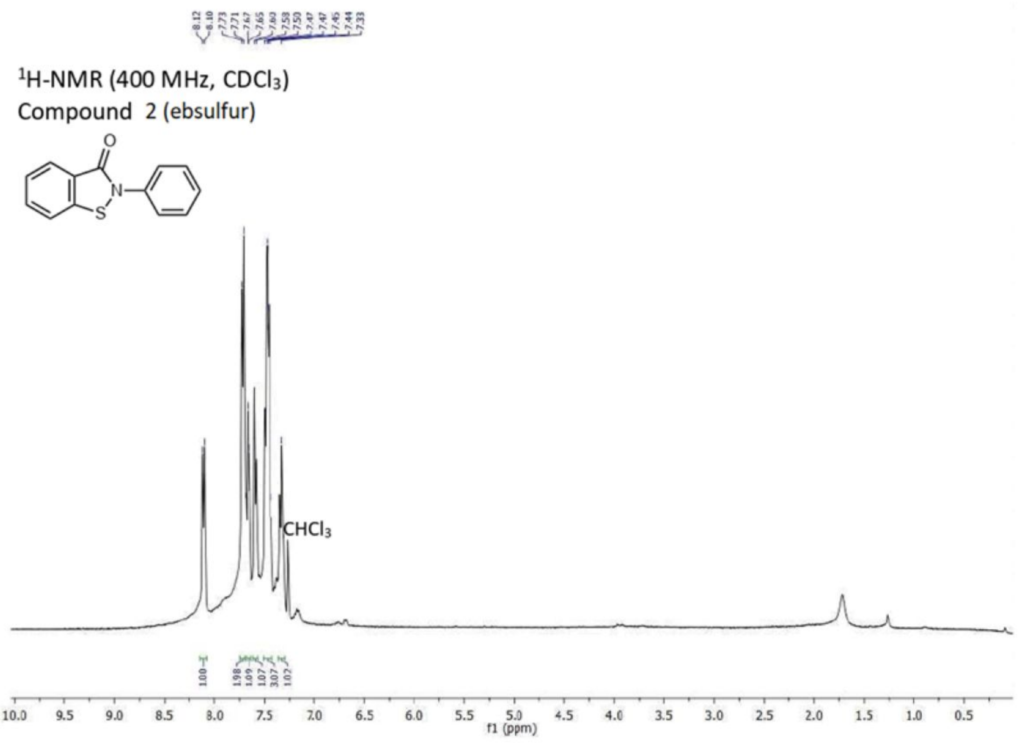

^{1}H-NMR (400 MHz, $CDCl_3$)
Compound 2 (ebsulfur)
$CHCl_3$
f1 (ppm)

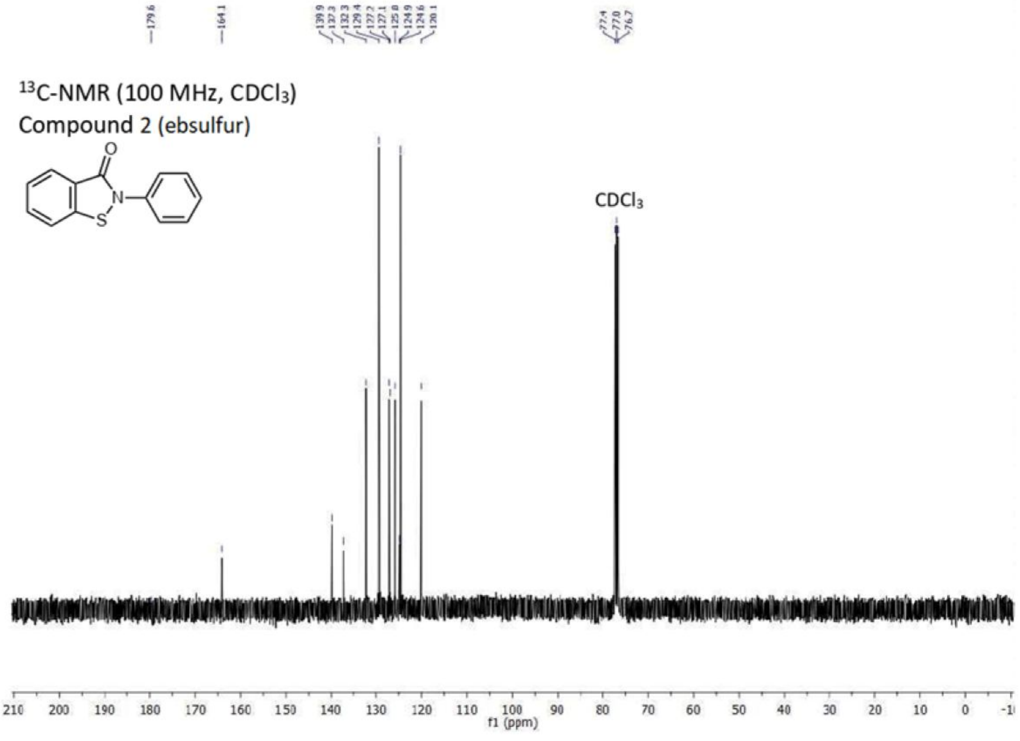

^{13}C-NMR (100 MHz, $CDCl_3$)
Compound 2 (ebsulfur)
$CDCl_3$
f1 (ppm)

Figure S7 E

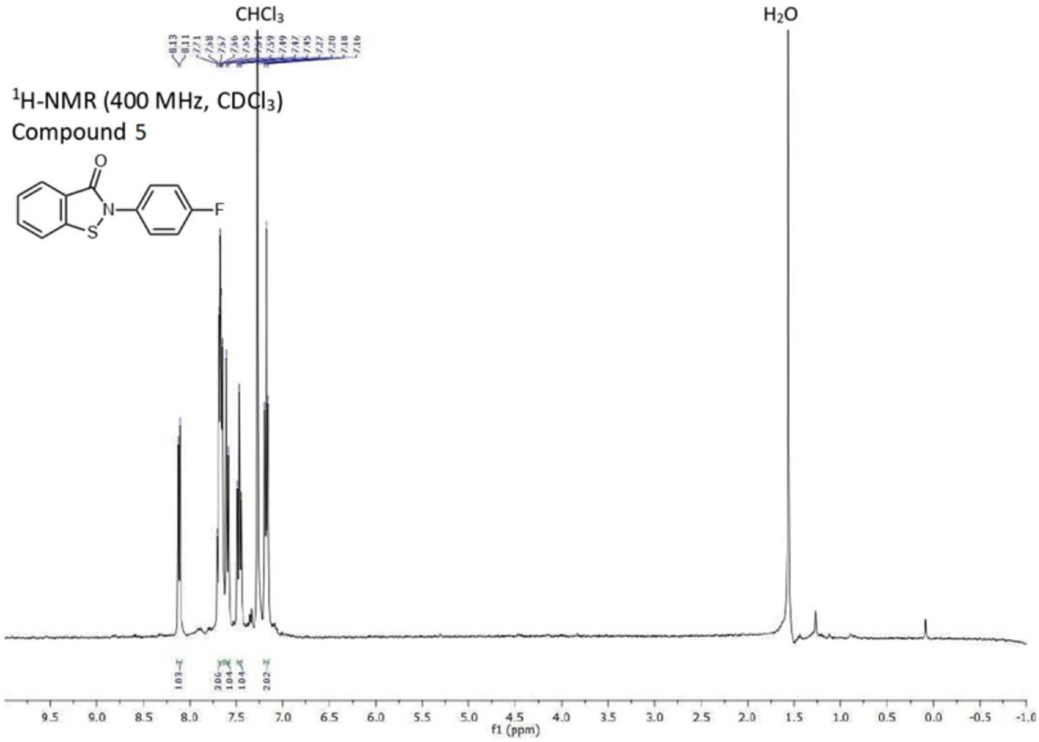
CHCl$_3$
H$_2$O
^{1}H-NMR (400 MHz, CDCl$_3$)
Compound 5
f1 (ppm)

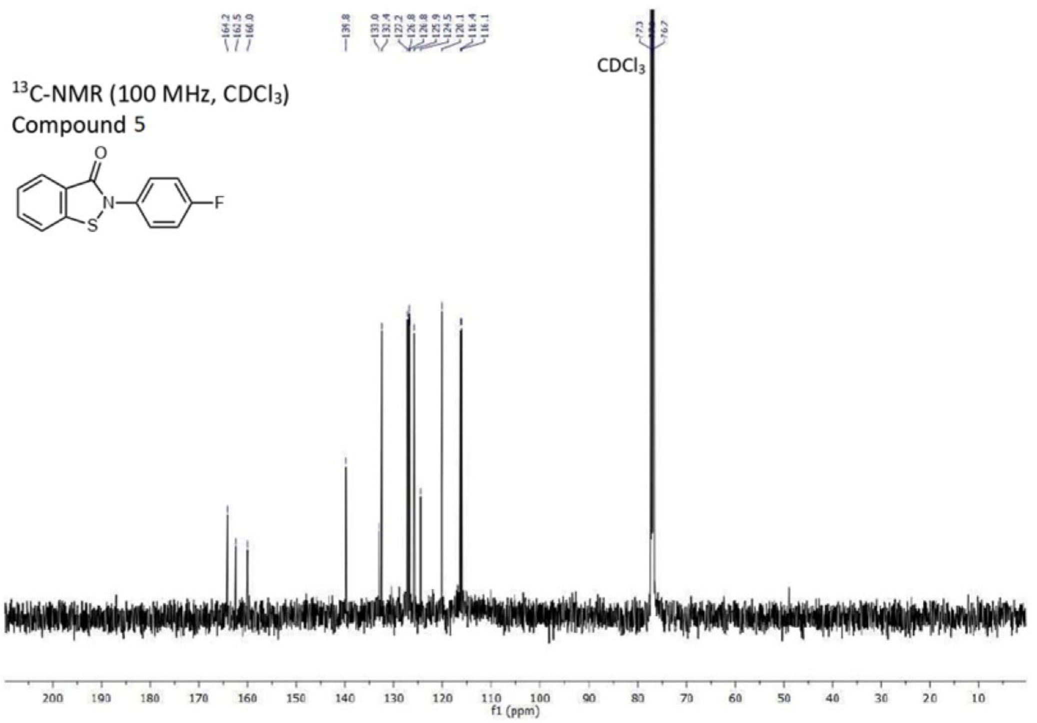
CDCl$_3$
^{13}C-NMR (100 MHz, CDCl$_3$)
Compound 5
f1 (ppm)

Figure S7 F

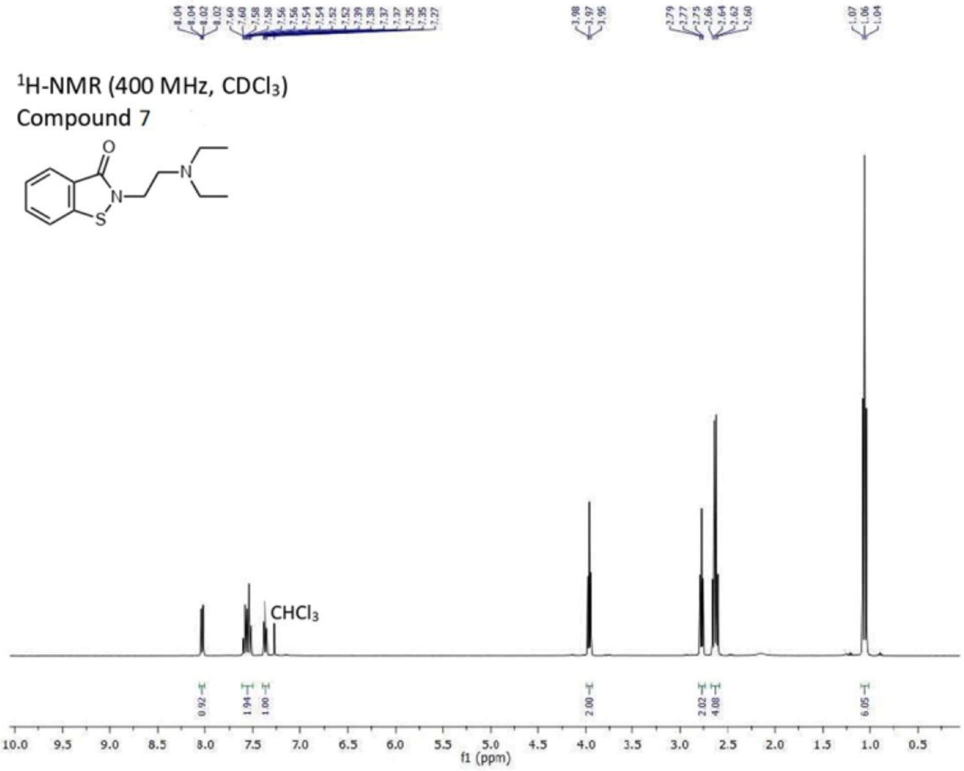
^{1}H-NMR (400 MHz, $CDCl_3$)
Compound 7
$CHCl_3$
f1 (ppm)

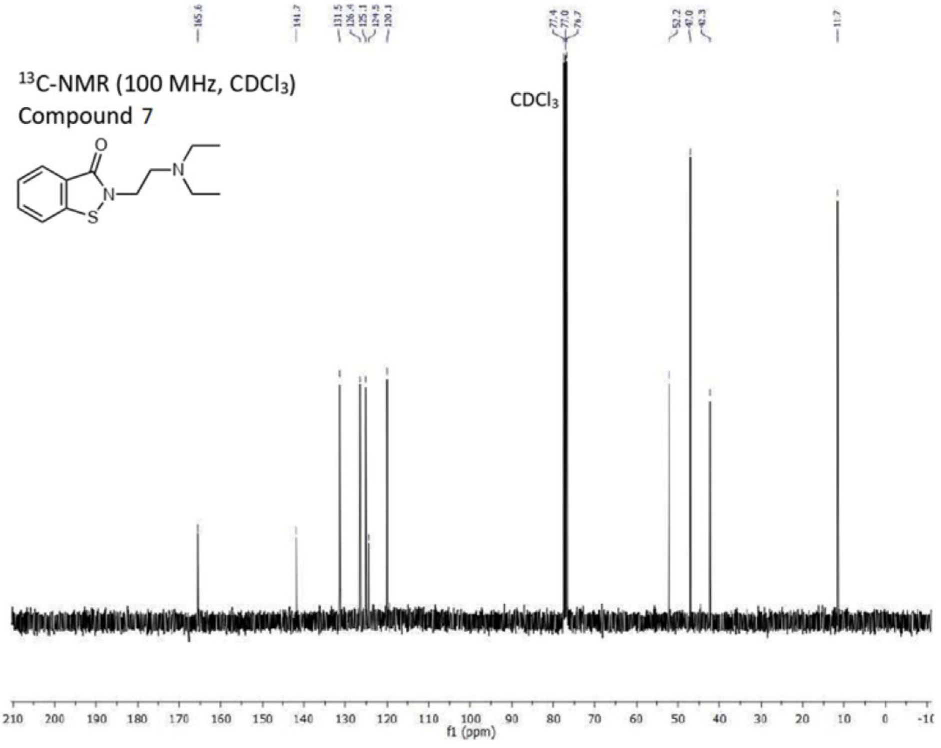
^{13}C-NMR (100 MHz, $CDCl_3$)
Compound 7
$CDCl_3$
f1 (ppm)

Figure S7 G

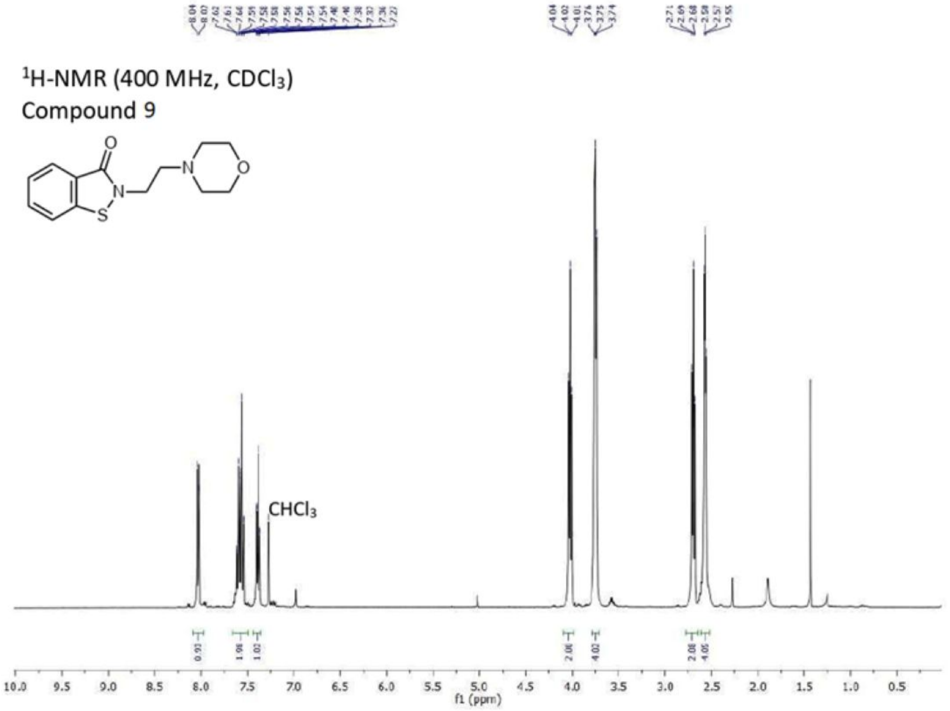

1H-NMR (400 MHz, CDCl3)
Compound 9
CHCl3
f1 (ppm)

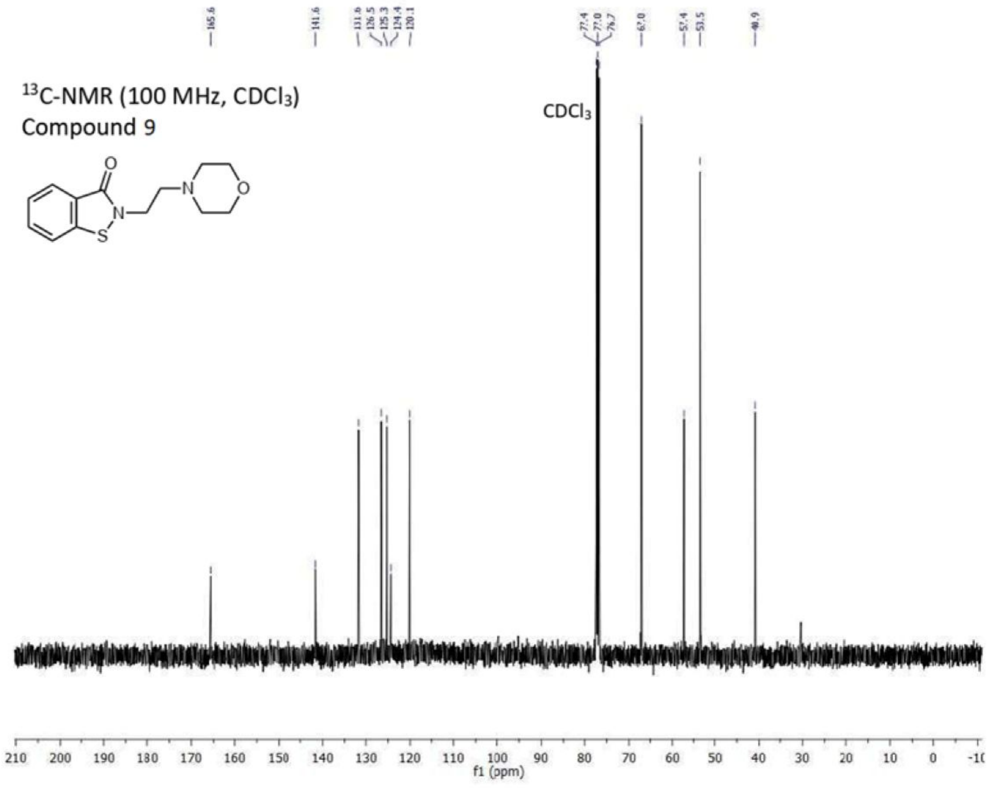

13C-NMR (100 MHz, CDCl3)
Compound 9
CDCl3
f1 (ppm)

Figure S7 H

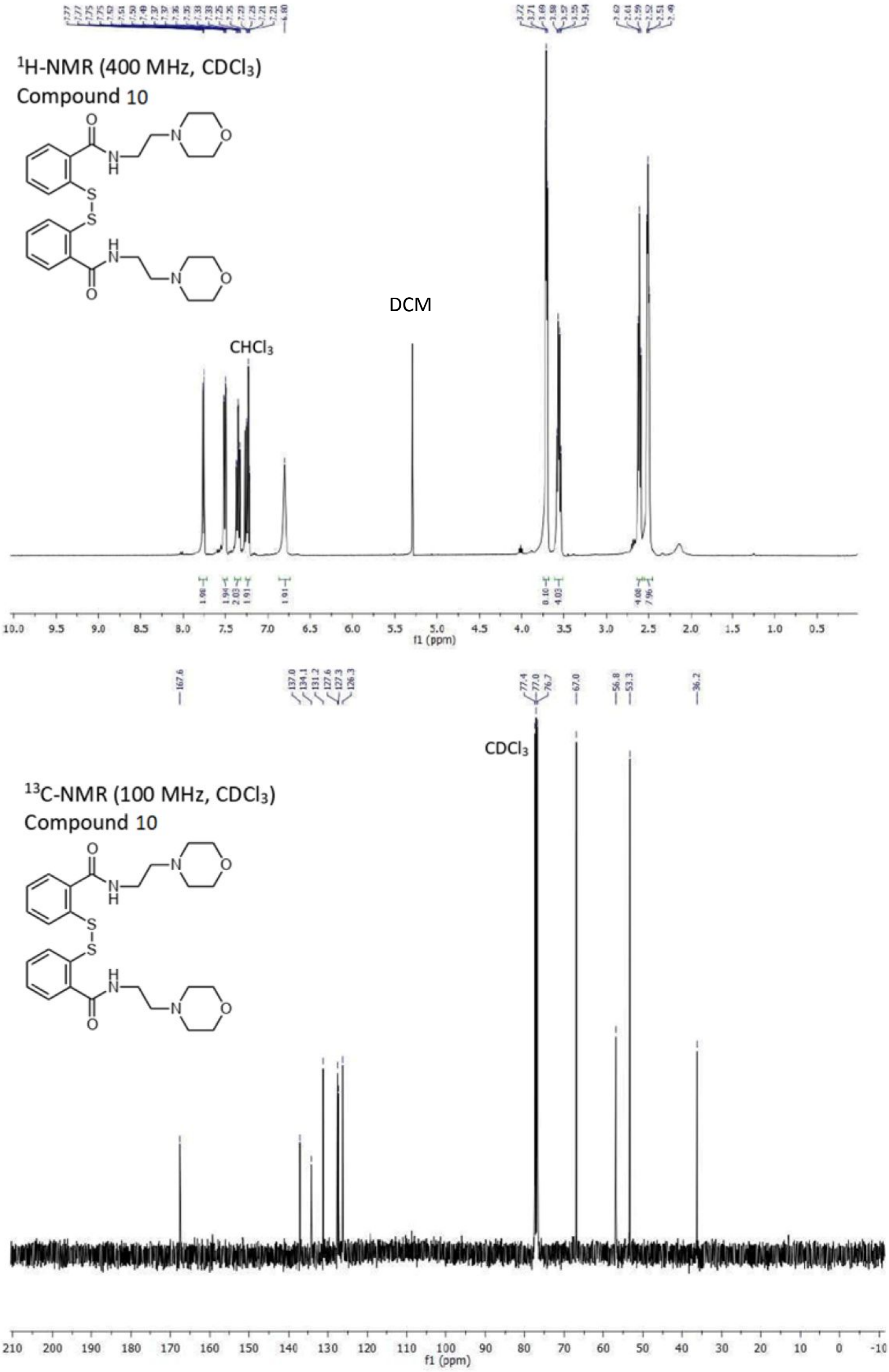

Supplementary Figure 7. NMR spectra of Ebselen analogues, A) compound 4, B) compound 2, C) compound 8, D) compound 6, E) compound 5, F) compound 7, G) compound 9, H) compound 10.

Figure S8 A

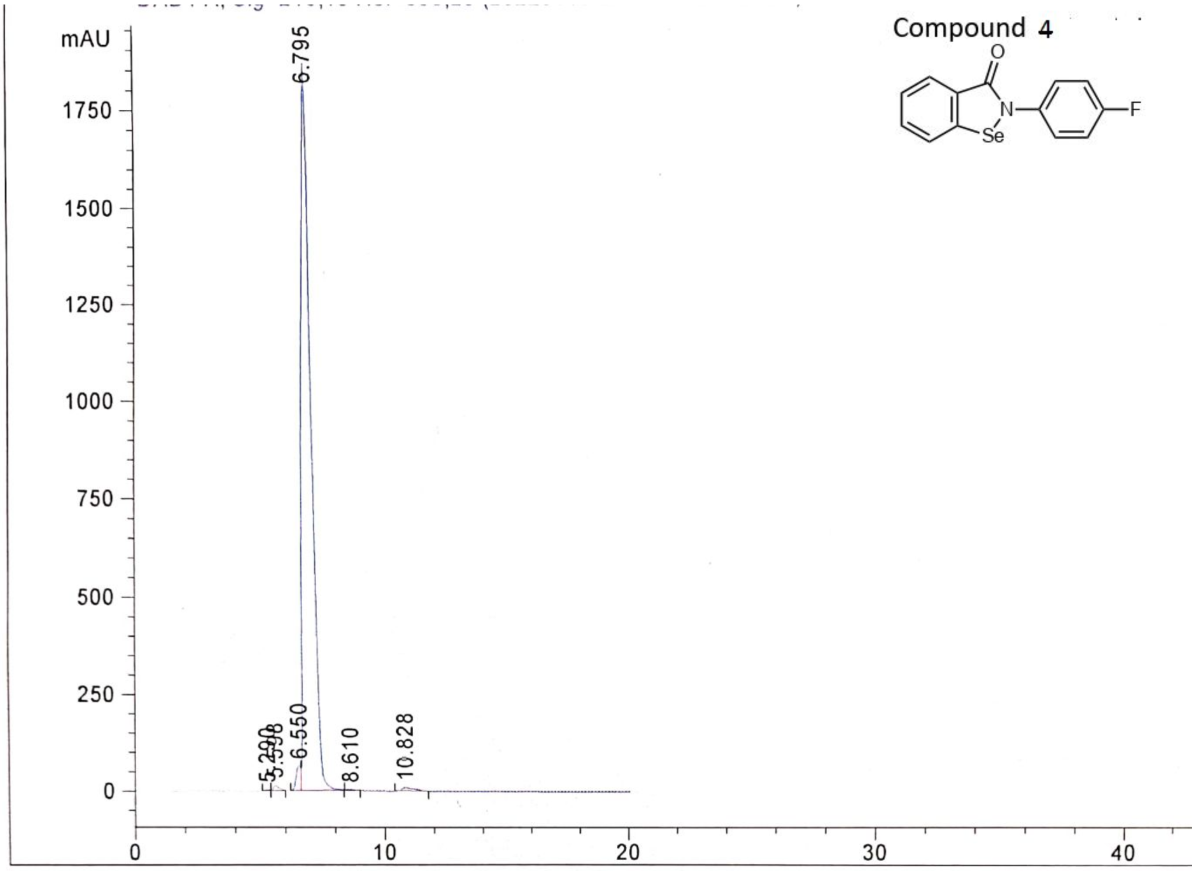

==
Area Percent Report
==

Sorted By : Signal
Multiplier : 1.0000
Dilution : 1.0000
Use Multiplier & Dilution Factor with ISTDs

Signal 1: DAD1 A, Sig=210,16 Ref=390,20

Peak #	RetTime [min]	Type	Width [min]	Area [mAU*s]	Height [mAU]	Area %
1	5.290	BV	0.1753	37.28167	3.21498	0.0751
2	5.598	VV	0.2145	235.59427	15.39898	0.4746
3	6.550	BV	0.2153	868.68274	65.24921	1.7499
4	6.795	VB	0.3401	4.80978e4	1870.00928	96.8883
5	8.610	BB	0.3624	72.32813	2.75364	0.1457
6	10.828	BB	0.4811	330.83414	9.52754	0.6664

Totals : 4.96426e4 1966.15362

Figure S8 B

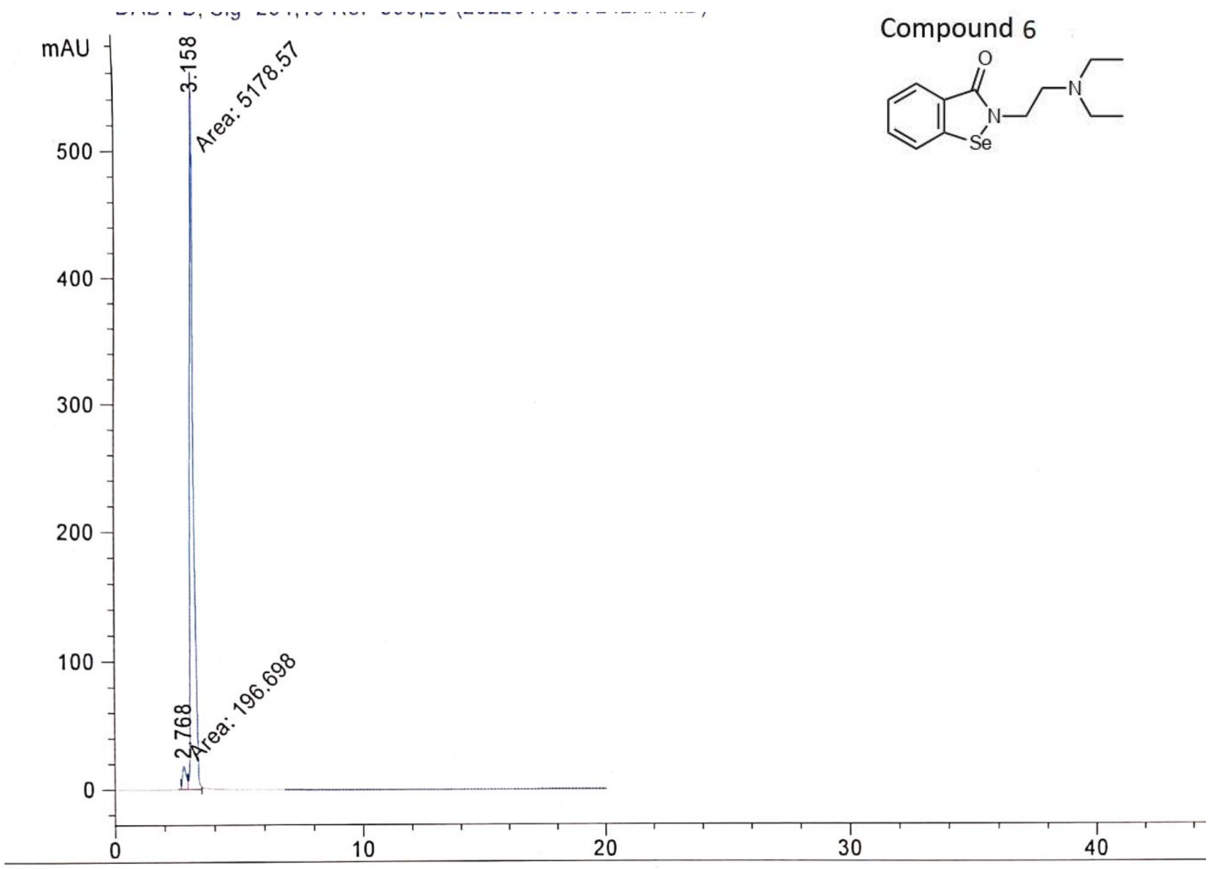

Compound 6

==

Area Percent Report

==

```
Sorted By                  :      Signal
Multiplier                 :      1.0000
Dilution                   :      1.0000
Use Multiplier & Dilution Factor with ISTDs
```

Signal 1: DAD1 B, Sig=254,16 Ref=390,20

Peak #	RetTime [min]	Type	Width [min]	Area [mAU*s]	Height [mAU]	Area %
1	2.768	MF	0.1758	196.69844	18.65268	3.6593
2	3.158	FM	0.1526	5178.57373	565.44519	96.3407

Totals : 5375.27217 584.09787

Figure S8 C

Compound 8

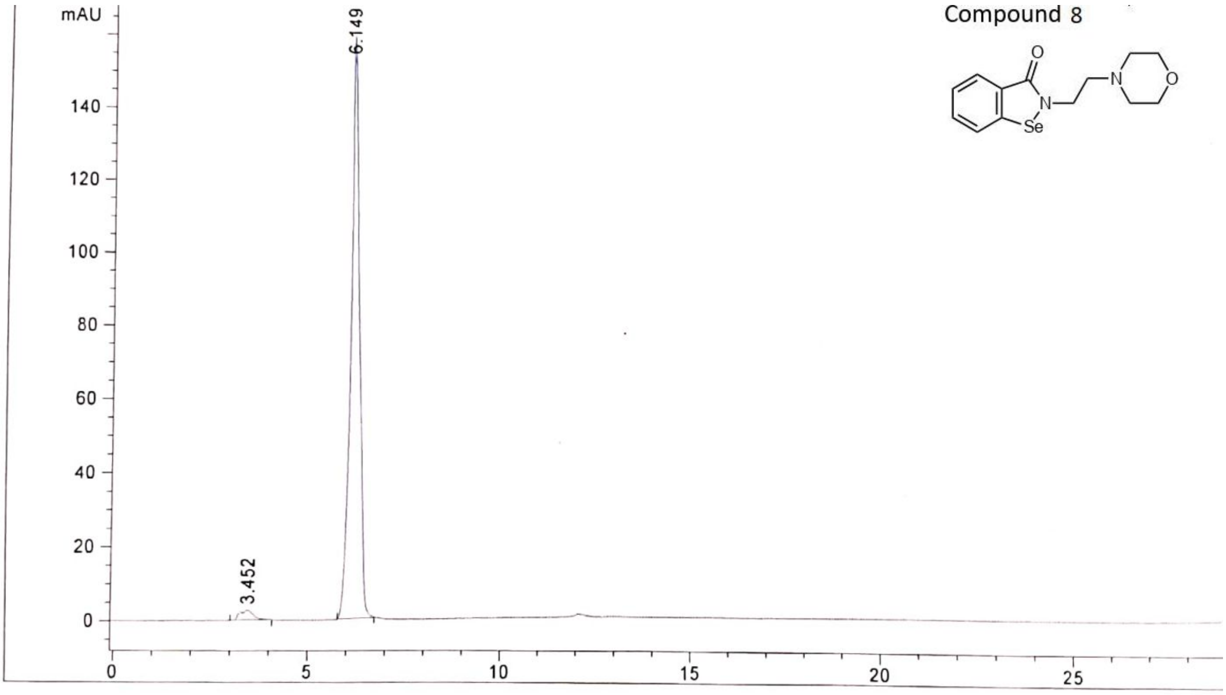


```
=====================================================================
                       Area Percent Report
=====================================================================

Sorted By              :      Signal
Multiplier             :      1.0000
Dilution               :      1.0000
Use Multiplier & Dilution Factor with ISTDs


Signal 1: DAD1 B, Sig=254,16 Ref=390,20
```

Peak #	RetTime [min]	Type	Width [min]	Area [mAU*s]	Height [mAU]	Area %
1	3.452	BB	0.3430	68.82885	2.79771	2.7359
2	6.149	BB	0.2143	2446.98145	160.09654	97.2641

Figure S8 D

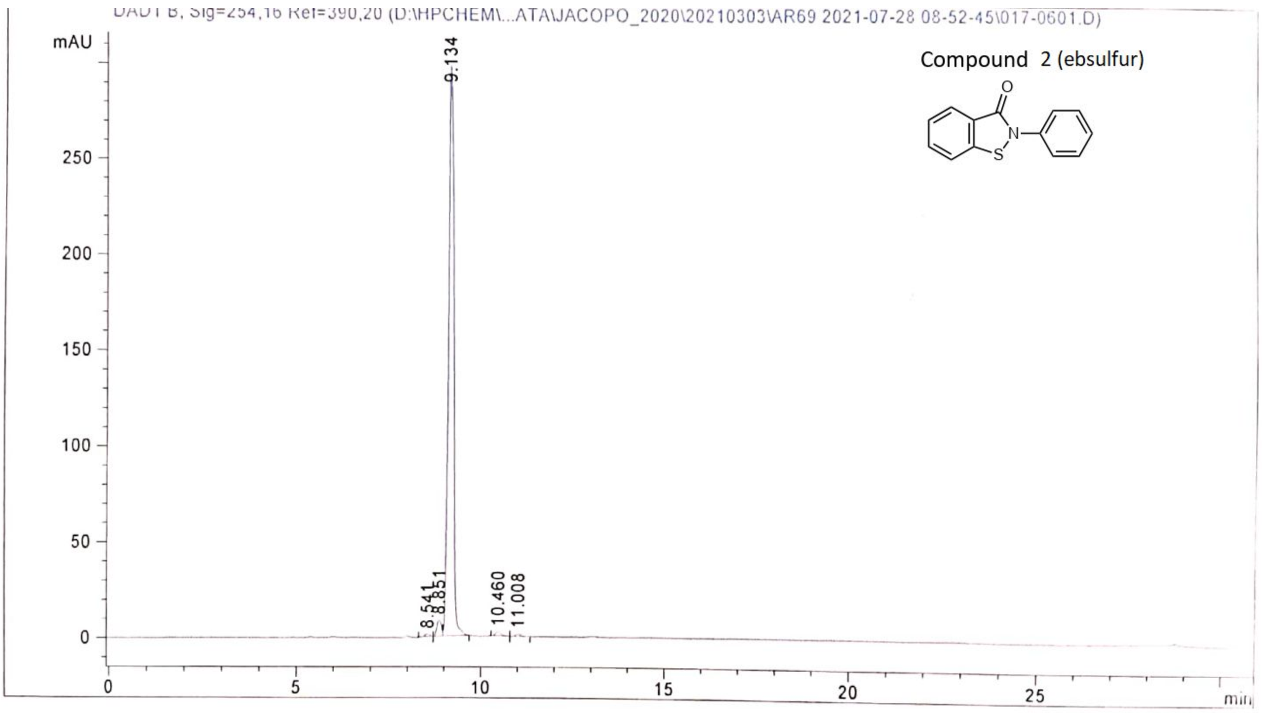


```
=====================================================================
                        Area Percent Report
=====================================================================

Sorted By             :      Signal
Multiplier            :      1.0000
Dilution              :      1.0000
Use Multiplier & Dilution Factor with ISTDs


Signal 1: DAD1 B, Sig=254,16 Ref=390,20
```

Peak #	RetTime [min]	Type	Width [min]	Area [mAU*s]	Height [mAU]	Area %
1	8.541	BV	0.1736	17.89709	1.66301	0.5688
2	8.851	VV	0.1467	80.19249	8.76839	2.5485
3	9.134	VB	0.1605	3002.78857	300.59180	95.4294
4	10.460	BB	0.1804	29.54241	2.52703	0.9389
5	11.008	BB	0.1678	16.18805	1.52592	0.5145

Figure S8 E

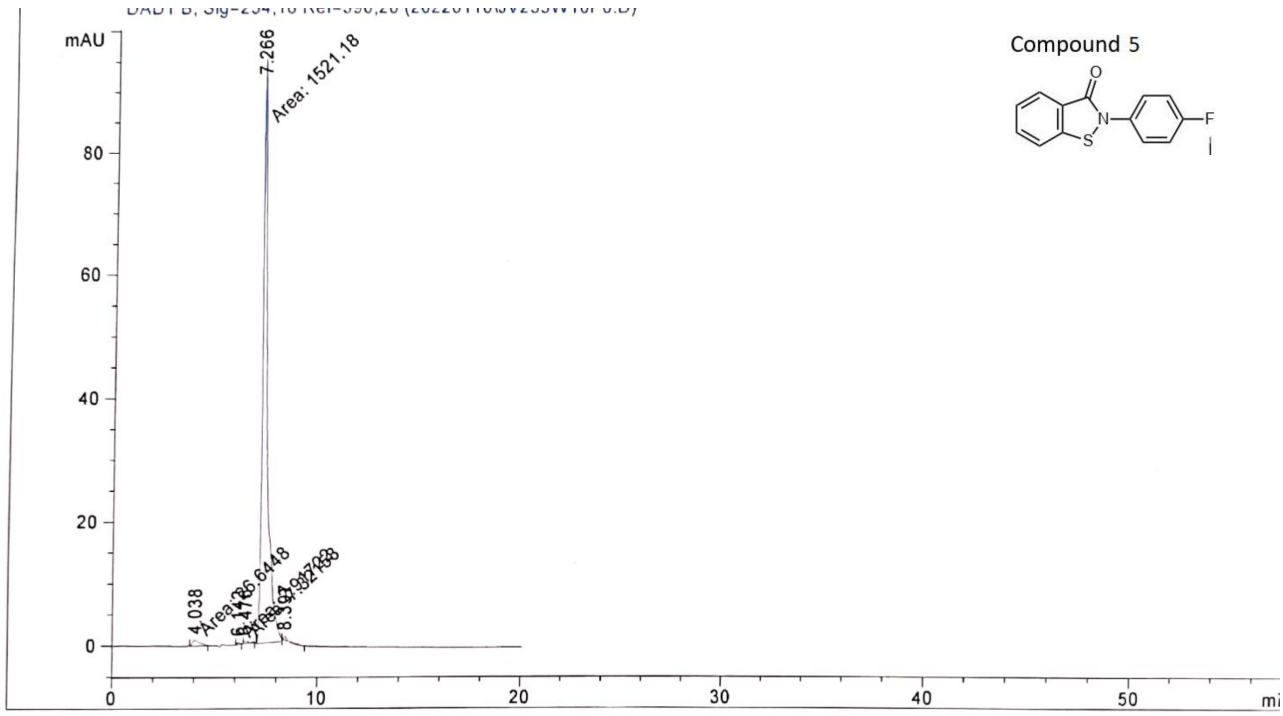

Compound 5

```
=================================================================
                    Area Percent Report
=================================================================

Sorted By             :      Signal
Multiplier            :      1.0000
Dilution              :      1.0000
Use Multiplier & Dilution Factor with ISTDs


Signal 1: DAD1 B, Sig=254,16 Ref=390,20
```

Peak #	RetTime [min]	Type	Width [min]	Area [mAU*s]	Height [mAU]	Area %
1	4.038	MM	0.4595	26.64482	9.66390e-1	1.7096
2	6.142	MM	0.1453	2.91722	3.34672e-1	0.1872
3	6.476	MM	0.3135	7.32158	3.89217e-1	0.4698
4	7.266	MM	0.2660	1521.17834	95.32454	97.6013
5	8.397	BB	0.0494	5.02014e-1	6.24825e-1	0.0322
Totals :				1558.56398	97.63964	

Figure S8 F

Compound 7

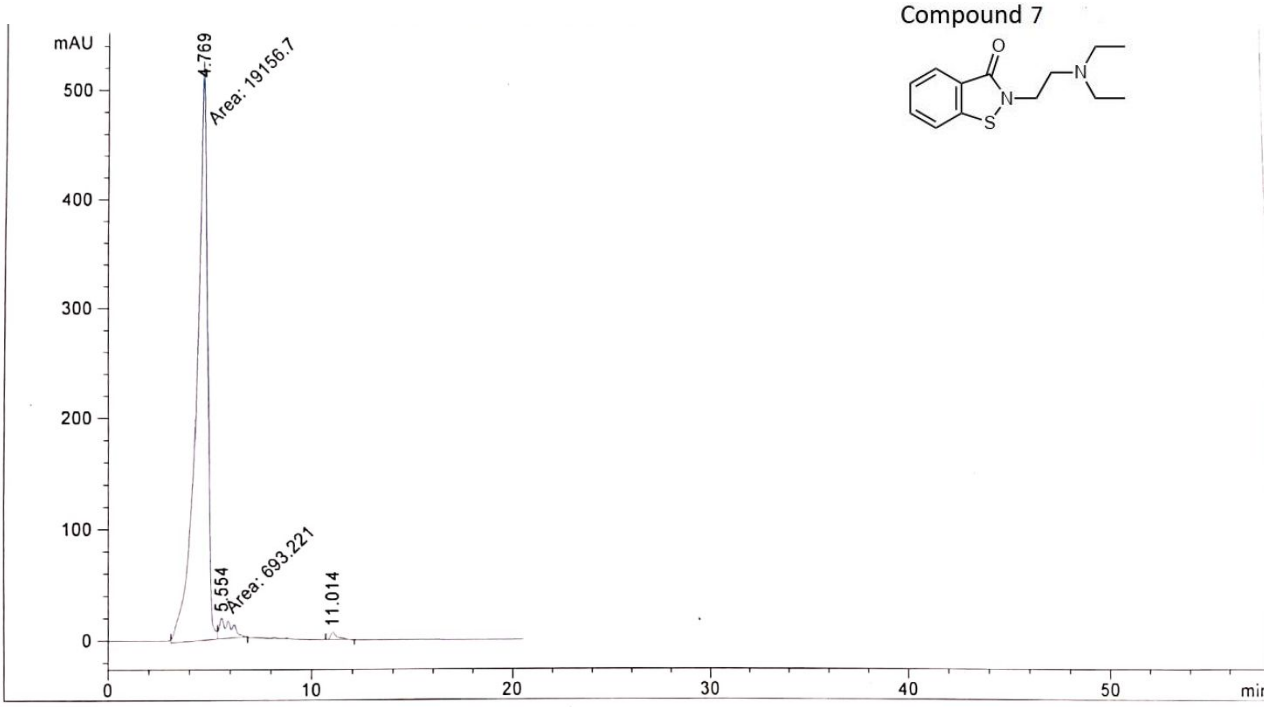

==

Area Percent Report

==

Sorted By : Signal
Multiplier : 1.0000
Dilution : 1.0000
Use Multiplier & Dilution Factor with ISTDs

Signal 1: DAD1 B, Sig=254,16 Ref=390,20

Peak #	RetTime [min]	Type	Width [min]	Area [mAU*s]	Height [mAU]	Area %
1	4.769	MF	0.6065	1.91567e4	526.43036	95.7333
2	5.554	FM	0.6036	693.22144	19.14192	3.4643
3	11.014	VB	0.3318	160.56773	6.79176	0.8024

Totals : 2.00105e4 552.36404

Figure S8 G

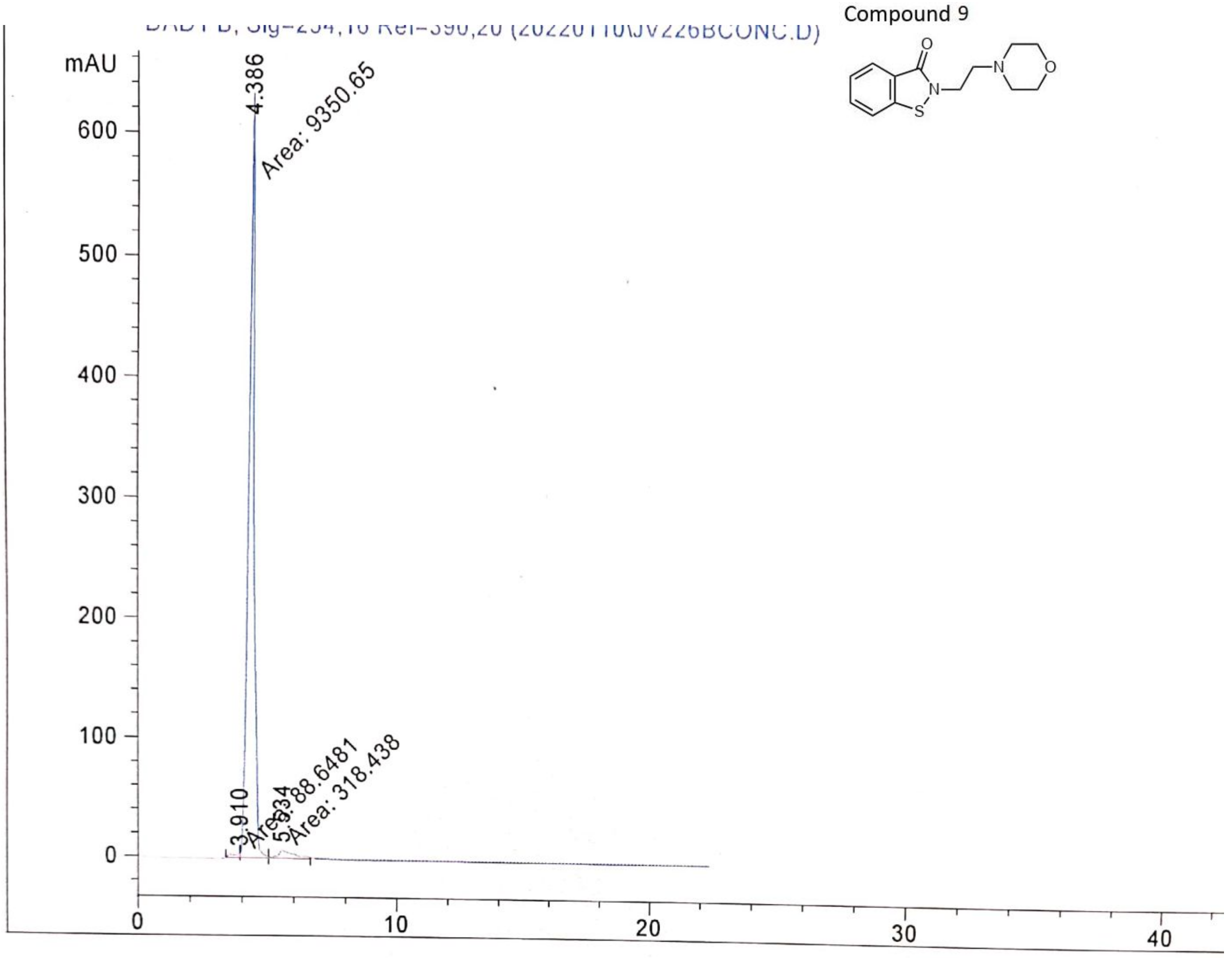

Compound 9

==

Area Percent Report

==

Sorted By : Signal
Multiplier : 1.0000
Dilution : 1.0000
Use Multiplier & Dilution Factor with ISTDs

Signal 1: DAD1 B, Sig=254,16 Ref=390,20

Peak #	RetTime [min]	Type	Width [min]	Area [mAU*s]	Height [mAU]	Area %
1	3.910	MF	0.3720	88.64809	3.97141	0.9085
2	4.386	MF	0.2450	9350.64746	636.16852	95.8281
3	5.534	MF	0.7106	318.43823	7.46870	3.2634

Totals : 9757.73379 647.60862

Figure S8 H

Compound 10

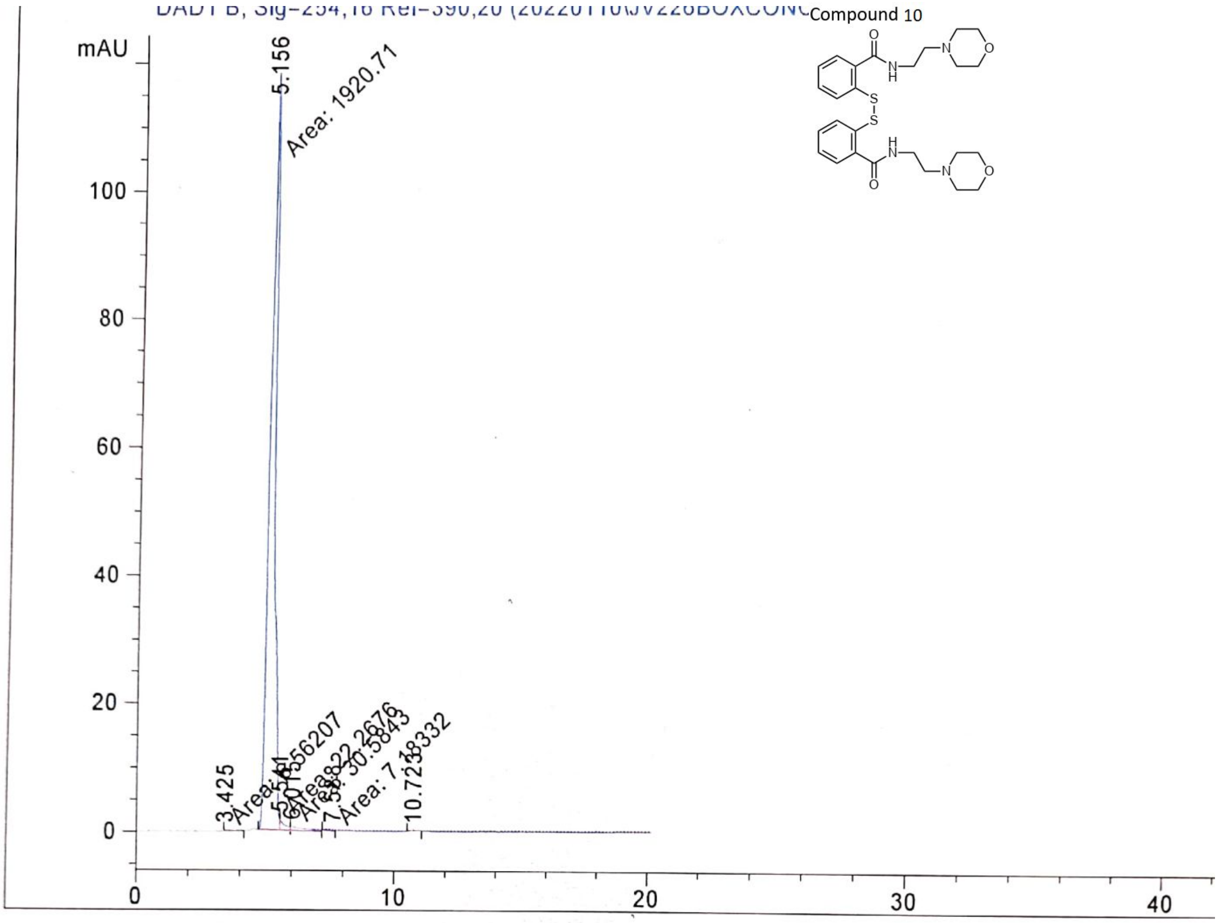

===

Area Percent Report

===

Sorted By : Signal
Multiplier : 1.0000
Dilution : 1.0000
Use Multiplier & Dilution Factor with ISTDs

Signal 1: DAD1 B, Sig=254,16 Ref=390,20

Peak #	RetTime [min]	Type	Width [min]	Area [mAU*s]	Height [mAU]	Area %
1	3.425	MM	0.4670	6.56207	2.34169e-1	0.3300
2	5.156	MF	0.2697	1920.70996	118.68906	96.5821
3	5.541	MF	0.2091	22.26765	1.74417	1.1197
4	6.015	FM	0.7073	30.58435	7.20714e-1	1.5379
5	7.588	MM	0.4445	7.18332	2.69313e-1	0.3612
6	10.723	BB	0.2176	1.37417	8.63845e-2	0.0691

Totals : 1988.68151 121.74381

Supplementary Figure 8. HPLC analysis of Ebselen analogues: A) compound **4** (RP18 isocratic MeOH/Water 9:1 flow 0.5 mL/min); B) compound **6** (RP18 isocratic MeOH/Water 9:1 flow 0.5 mL/min); C) compound **8** (RP18 gradient ACN/Water 3:7 to 8:2 flow 1 mL/min); D) compound **2** (RP18 gradient ACN/Water 3:7 to 8:2 flow 1 mL/min); E) compound **5** (RP18 isocratic MeOH/Water 9:1 flow 0.5 mL/min); F) compound **7** (RP18 isocratic MeOH/Water 9:1 flow 0.5 mL/min); G) compound **9** (RP18 isocratic MeOH/Water 9:1 flow 0.5 mL/min); H) compound **10** (RP18 isocratic MeOH/Water 9:1 flow 0.5 mL/min).
